# Formation of amides: one-pot condensation of carboxylic acids and amines mediated by TiCl_4_

**DOI:** 10.1186/s13065-017-0318-9

**Published:** 2017-09-15

**Authors:** Antonella Leggio, Jessica Bagalà, Emilia Lucia Belsito, Alessandra Comandè, Marianna Greco, Angelo Liguori

**Affiliations:** Dipartimento di Farmacia e Scienze della Salute e della Nutrizione, Università della Calabria Edificio Polifunzionale, 87036 Arcavacata, CS Italy

**Keywords:** Amides, Titanium tetrachloride, Carboxylic acids, Amines, Condensation reaction

## Abstract

A general procedure for the synthesis of amides via the direct condensation of carboxylic acids and amines in the presence of TiCl_4_ is reported. The amidation reaction was performed in pyridine at 85 °C with a wide range of substrates providing the corresponding amide products in moderate to excellent yields and high purity. The reaction proceeds with low yields when both the carboxylic acid and the amine are sterically hindered. The process takes place with nearly complete preservation of the stereochemical integrity of chiral substrates.

**Electronic supplementary material:**

The online version of this article (doi:10.1186/s13065-017-0318-9) contains supplementary material, which is available to authorized users.

## Introduction

Amide is a key functional group in organic chemistry for its widespread occurrence in peptide and non-peptide natural products, therapeutic small molecules, and new polymeric materials [1–4].

The most general way for obtaining amides involves the activation of the carboxylic function by means the conversion of carboxylic acids into the corresponding acid chlorides [5–8]. Subsequently this reactive derivative is coupled with the appropriate amine to yield the corresponding amide.

Alternatively, carboxylic acids, by the use of activating reagents, can be transformed into reactive acylating intermediates (acyl chlorides, anhydrides, activated esters) which directly react in situ with the suitable amines without their preliminary isolation and purification [9–12].

The use of coupling reagents is the only practicable way when the reagents useful for obtaining acid chlorides from carboxylic acids are not compatible with other chemical functions or protecting groups present on the substrate.

The importance of amides has promoted the development of new protocols and reagents based on these approaches and alternative methods for amide bond formation [13–16].

The direct formation of amides by condensing non-activated carboxylic acids and amines is extremely attractive because of its low environmental impact. Using metal-based catalysis in direct amide preparation, as an alternative to coupling reagents, has been reported [17–19].

The main synthetic catalysts employed for direct amidation are boronic acids and esters together with Lewis acid metal complexes.

Boron-based compounds are reported as catalysts promoting the condensation of carboxylic acids and amines in refluxing toluene [20, 21].

In addition amidation reaction protocols by using boronic acid and ester catalysts were also developed for the formation of dipeptide systems [22–24].

Under homogeneous conditions, the most used metal catalysts for direct coupling of carboxylic acids with amines are group IV metals.

In 1972 Werdehausen et al. [25] developed a catalytic procedure for the direct formation of amides starting from different long chain fatty acids and amines by using 0.6–1 mol% of metal catalysts based on Ti(IV), Zr(IV) and Ta(V), at 120–200 °C.

Later carboxyamides from benzoic acids and different amines were obtained by using stochiometric amounts of various Lewis acid catalysts in refluxing toluene [26].

Ti(OBu)_4_ was used in catalytic amounts for obtaining amides from several carboxylic acids and anilines in refluxing o-xylene with yields ranging from 38 to 98%, electron donating groups on the aromatic ring of anilines provided higher conversions than electron withdrawing substituents [27, 28].

Different amides were synthesized using titanium(IV) isopropoxide as catalyst in 10–20 mol% loading in THF [29]. Electronically and sterically demanding substrates provided their corresponding amides in moderate to good yields using a reaction temperature of 100 °C. The hydrolytic decomposition of the catalyst caused a drastic lowering of the reaction yields when the reaction was performed in air.

The use of Zirconium(IV) catalysts for the direct amidation of carboxylic acids and amines was also reported [17]. ZrCl_4_ and ZrCp_2_Cl_2_ were particularly effective resulting in high conversions of the substrates after 4 h of reaction time at 110 °C using a 5 mol% catalyst.

Simultaneously, Adolfsson et al. developed a ZrCl_4_ catalysed amidation protocol at 70 °C in THF with molecular sieves as water scavengers [30, 31]. Both aliphatic and aromatic carboxylic acids were converted in secondary and tertiary amides in 62–99% yield with 2–10 mol% catalyst loading. Aromatic carboxylic acids required reaction temperatures of 100 °C and 10% catalyst loading in order to increase the yields after 24 h reaction time.

The sandwich complex bis(dicyclopentadienyl)hafnium dichloride ([Hf(Cp)2Cl2]) was used as catalyst for direct amidation of nonactivated carboxylic acids at 26 °C [32]. The method provided different amides in a reaction time of 48 h and using a 10% catalyst loading. Some substrates were converted in the corresponding amides before this time.

In our research activity, in which titanium tetrachloride was employed, it was observed the oxophilic character of this transition metal towards carbonyl and hydroxyl groups [33–38].

On the base of these observations, we became interested in investigating the direct coupling of carboxylic acids and amines using TiCl_4_ as condensing agent.

Here, we report the successful TiCl_4_-mediated synthesis of secondary and tertiary amides starting from various carboxylic acid and amine precursors.

## Results and discussion

Optimization of amidation reaction conditions was performed by choosing benzoic acid as model substrate.

The reaction was previously investigated under catalytic conditions. Benzoic acid (1 mmol) and a catalytic amount of TiCl_4_ (30 mol%) were treated with aniline (1 mmol) in refluxing dry dichloromethane. During the addition of TiCl_4_ to the carboxylic acid, the production of hydrochloric acid was observed. After 12 h of reaction small amounts of amide (12%) were obtained. Then the reaction was repeated for a longer reaction time (24 h). Also in this case the reaction did not proceed and the amide product was recovered with low yield (15%). The poor outcome of the reaction prompted us to use a base to covert the carboxylic acid into the corresponding carboxylate. Pyridine was chosen as base to form the pyridinium carboxylate. It was not possible to use non-nucleophilic tertiary nitrogen bases such as triethylamine for the known reactivity of the trialkyl amines with TiCl_4_ [39, 40]. The reaction was designed in dichloromethane excluding ethereal solvents such as THF because, in the presence of TiCl_4_, O-heterocycle ring opening reaction occurs [41, 42]. Therefore the amidation reaction was carried out in dichloromethane under reflux by treating benzoic acid (1 mmol) with pyridine (1 mmol) and TiCl_4_ (1.5 mmol), then aniline was added (1 mmol). In the reaction mixture, during the adding of pyridine the formation of an insoluble salt, probably relating to the pyridinium salt, was observed. After a reaction time of 12 h benzanilide was recovered in 38% yield.

In the light of these results it was chosen to carry out the reaction in pyridine, which performs the function of solvent and base also by neutralizing the hydrochloric acid that develops during the reaction, using a higher temperature and an excess of TiCl_4_ to accelerate the reaction.

Benzoic acid (1 mmol) was dissolved in pyridine (10 mL) in a screw capped vial. Then, to the resulting solution heated at 85 °C, were added TiCl_4_ (3 mmol) and aniline (1 mmol). After about 2 h reacting, the reaction was completed and the reaction mixture was acidified with 1 N HCl aqueous solution and extracted with methylene chloride. The organic phase provided the N-phenylbenzamide in 98% yield and high purity as confirmed by GC/MS and NMR analyses.

Previously, TiCl_4_ was used in stoichiometric amounts to form carboxamides from carboxylic acids [18]. In this case the reaction was performed in THF or hexane as solvent and the nucleophilic amine was used in large excess to neutralize the hydrochloric acid produced during the reaction. The reported procedure is poorly applicable as the reaction times are very long (8 h–7 days) also when heating is required.

Our result was particularly good and interesting, as the product was recovered pure in short times and in excellent yield without requiring the use of complex purification procedures.

**Scheme 1:**
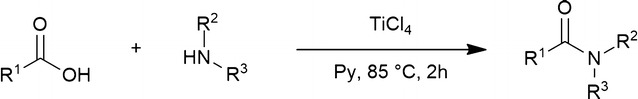
Direct formation of amides assisted by TiCl_4_

Encouraged by the success of this preliminary study, the adopted procedure was applied for the preparation of a series of *N*-phenylamides. Different alkyl and aryl carboxylic acids were tested under the developed reaction conditions using aniline, the amine component, as a constant (Scheme 1).

Aliphatic and aromatic carboxylic acids performed well, delivering the desired amides (**1**–**7**) in excellent yields (entry **a**–**g**, Table 1).

**Table 1:** Synthesis of *N*-phenylamides **1**−**7**

Entry	R^1^	R^2^	R^3^		Product	Yield (%)^a^
**a**	C_6_H_5_	H	C_6_H_5_	**1**		98
**b**	4-NO_2_–C_6_H_4_	H	C_6_H_5_	**2**		98
**c**	4-CH_3_O–C_6_H_4_	H	C_6_H_5_	**3**		95
**d**	4-Cl–C_6_H_4_	H	C_6_H_5_	**4**		95
**e**	C_6_H_5_CH_2_	H	C_6_H_5_	**5**		95
**f**	C_6_H_5_CH=CH	H	C_6_H_5_	**6**		91
**g**	C_15_H_31_	H	C_6_H_5_	**7**		88

^a^Isolated yields

The molecular structure of the obtained amides was assigned by ^1^H NMR, ^13^C NMR and GC/MS analyses.

Additional experiments, that employed 2-fluoroaniline and 4-methylaniline as amine components, were carried out in order to investigate the electronic effect of the substituent on the aniline nucleophilicity and consequently on the reaction course. The reactions went to completion in short times (2 h) and afforded the corresponding amides **8**–**9** in high yields (Scheme 1; Table 2).

**Table 2:** Synthesis of substituted anilides **8**−**9**

Entry	R^1^	R^2^	R^3^		Product	Yield (%)^a^
**h**	C_6_H_5_CH_2_	H	2-F–C_6_H_4_	**8**		72
**i**	C_6_H_5_CH_2_	H	4-CH_3_–C_6_H_4_	**9**		98

^a^Isolated yields

The lower nucleophilicity of 2-fluoroaniline resulted in a lower yield of the corresponding amide (**8**) (72%, Table 2).

We explored also the reactivity of the more nucleophilic alkyl amines by choosing, as amine substrate, the propylamine. The standard protocol applied to the reaction of propylamine with aliphatic and aromatic carboxylic acids provided the corresponding amides **10**–**16** with yields higher than 90% (Scheme 1; Table 3).

**Table 3:** Synthesis of *N*-propylamides** 10**–**16**

Entry	R^1^	R^2^	R^3^		Product	Yield (%)^a^
**j**	C_6_H_5_	H	C_3_H_7_	**10**		91
**k**	4-NO_2_–C_6_H_4_	H	C_3_H_7_	**11**		92
**l**	4-CH_3_O–C_6_H_4_	H	C_3_H_7_	**12**		78
**m**	4-Cl–C_6_H_4_	H	C_3_H_7_	**13**		96
**n**	C_6_H_5_CH_2_	H	C_3_H_7_	**14**		95
**o**	C_6_H_5_CH=CH	H	C_3_H_7_	**15**		97
**p**	C_15_H_31_	H	C_3_H_7_	**16**		94

^a^Isolated yields

Amide formation, by using the developed reaction protocol, was also studied with disubstituted amines that are often more difficult substrates since they exhibit a significant steric hindrance.

Diethylamine was chosen as a representative dialkyl amine for studying the influence of steric hindrance on the course of the reaction (Scheme 1; Table 4).

**Table 4:** Synthesis of *N*,*N*-diethylamides **17**–**23**

Entry	R^1^	R^2^	R^3^		Product	Yield (%)^a^
**q**	C_6_H_5_	C_2_H_5_	C_2_H_5_	**17**		64
**r**	4-NO_2_–C_6_H_4_	C_2_H_5_	C_2_H_5_	**18**		80
**s**	4-CH_3_O–C_6_H_4_	C_2_H_5_	C_2_H_5_	**19**		56
**t**	4-Cl–C_6_H_4_	C_2_H_5_	C_2_H_5_	**20**		77
**u**	C_6_H_5_CH_2_	C_2_H_5_	C_2_H_5_	**21**		85
**v**	C_6_H_5_CH=CH	C_2_H_5_	C_2_H_5_	**22**		87
**w**	C_15_H_31_	C_2_H_5_	C_2_H_5_	**23**		91

^a^Isolated yields

The desired tertiary *N*,*N*-diethylamides were obtained with satisfactory yields even though lower than secondary amides probably due to the steric hindrance of diethylamine (Table 4).

The results reported in Table 4 also suggested that, when diethyl amine is used as amine component, the reaction is affected by the electronic nature of the substituent on the aromatic ring of the benzoic acid (entry **r**, entry **t** Table 4) which probably characterize the reactivity of the reaction intermediates.

In fact, the presence of chlorine and nitro group on the aromatic ring of carboxylic acids results in higher yields in amide (**18**, **20**, Table 4) than substrates that have no substituents or have electron donor groups on the aromatic ring. (**17**, **19**, Table 4). This is consistent with the higher reactivity of the involved intermediates in the formation of **18** and **20**.

In many experiments in which benzoic acid was used, such as in the synthesis of *N*,*N*-diethylbenzamide (**17**), traces of benzoyl chloride were found in the crude product. This observation suggested that the acyl chloride could be the reaction intermediate.

**Scheme 2:**
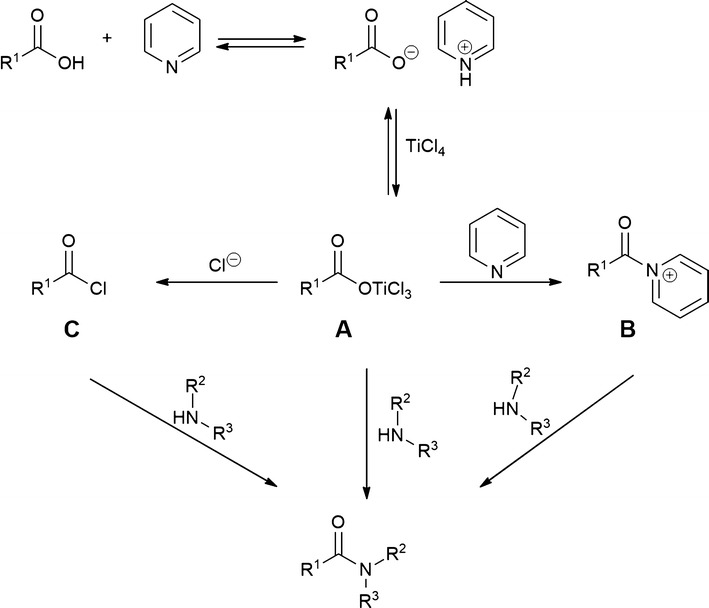
Proposed mechanism for the TiCl_4_ assisted direct amidation

The amidation reaction could proceed through the formation of an adduct (**A**) between the carboxylate ion, generated by the reaction of the carboxylic acid with pyridine, and TiCl_4_ (Scheme 2) as reported in literature with other metals [30]. The adduct **A** is characterized by the presence of a good leaving group that could lead to: (a) the direct formation of the amide; (b) the formation of the acyl pyridinium ion (**B**); (c) the formation of the acyl chloride (*C*) (Scheme 2). **B** and **C** could act as additional reaction intermediates for the formation of the amide.

The reaction of the sterically hindered pivalic acid with diethylamine resulted with very low conversions and provided, after 2 h, the corresponding amide **26** in 9% yield. (entry **z**, Table 5). When the reaction was performed by treating pivalic acid with aniline and propylamine (entry **x**–**y**, Table 5), the amides **24** and **25** were recovered respectively with 90 and 75% yield (Table 5) demonstrating that the steric effect of the carboxylic acid alone has minor impact on the reaction course.

**Table 5:** Synthesis of pivalic acid amides **24**–**26**

Entry	R^1^	R^2^	R^3^		Product	Yield (%)^a^
**x**	(CH_3_)_3_C	H	C_6_H_5_	**24**		90
**y**	(CH_3_)_3_C	H	C_3_H_7_	**25**		75
**z**	(CH_3_)_3_C	C_2_H_5_	C_2_H_5_	**26**		9

^a^Isolated yields

In these reactions, steric effects play a key role when both amine and carboxylic acid are sterically hindered.

**Scheme 3:**
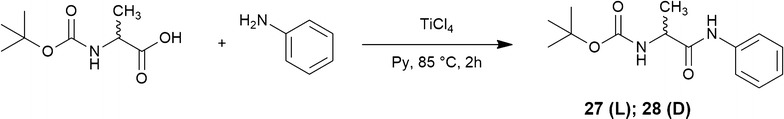
Direct formation of enantiomeric amides **27**–**28** assisted by TiCl_4_

We also explored the stereochemical outcome of the adopted procedure by synthesizing a couple of anilide enantiomers **27** and **28** starting from (*S*)-2-(*N*-*tert*-butoxycarbonylamino)propanoic acid (*N*-Boc-l-Alanine) and (*R*)-2-(*N*-*tert*-butoxycarbonylamino)propanoic acid (*N*-Boc-d-Alanine) as acid substrates respectively (Scheme 3). Gratifyingly, we were able to synthesize the two enantiomers with high enantioselectivity.

*N*-Boc-l-alanine and *N*-Boc-d-alanine reacted with aniline in the presence of TiCl_4_ affording the corresponding amides **27** and **28** respectively in 88 and 87%  % yield (Scheme 3).

This result demonstrates that this reaction works well also with carboxylic acids bearing acid-labile protecting groups.

**Fig. 1:**
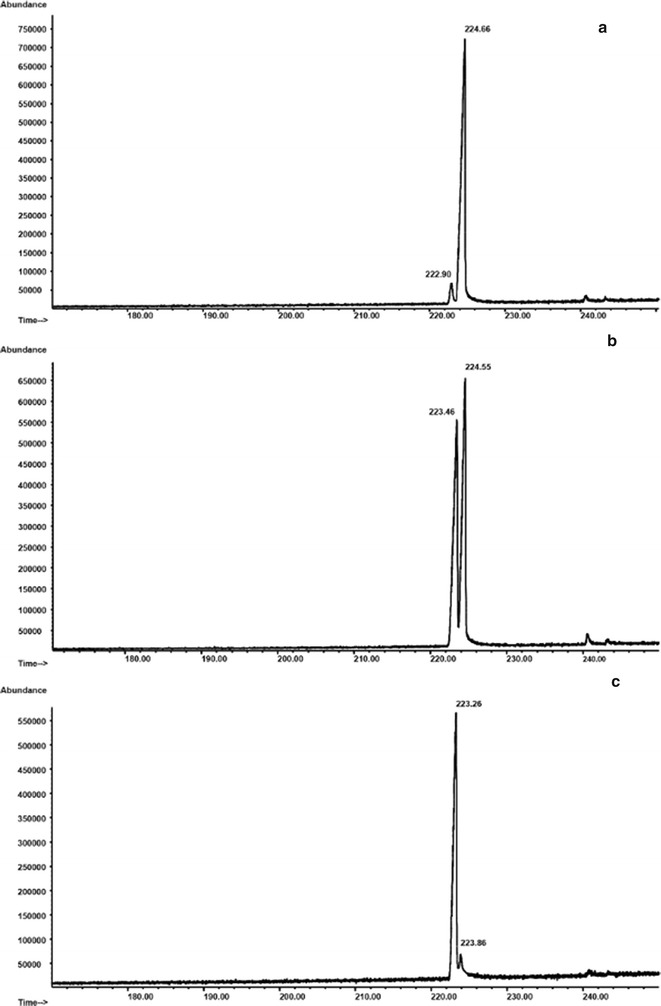
Chiral GC/MS analyses of crude amides **27** and **28**: **a** (S)-2-(*N*-*tert*-butoxycarbonyl-amino)-*N*-phenylpropanamide (**27**); **b** a mixture of **27** and **28** (approx. 1:1); **c** (R)-2-(*N*-*tert*-butoxycarbonylamino)-*N*-phenylpropanamide (**28**)

The enantiomeric purity of each crude product **27** and **28** was assessed by analyzing them by chiral GC/MS and comparing the resulting chromatograms with that of a suitably prepared mixture (approx. 1:1) of the two enantiomers (Fig. 1). The two enantiomers of the mixture, after finding the optimal conditions for achieving good selectivity, were resolved by chiral GC/MS: two peaks with retention times of 223.46 and 224.55 min corresponding to **28** and **27** respectively appeared in the chromatogram (Fig. 1b).

The comparison of the chromatograms of the single crude enantiomers **27** and **28** (Fig. 1a, c) with the mixture and the integration gave the data for the enantiomeric excess (ee) calculation, which was 95% for the d-isomer and 93% for the L-isomer. The calculated enantiomeric excess was for both enantiomers satisfactory.

## Conclusion

A general approach to generate amides has been established using TiCl_4_-induced direct condensation of carboxylic acids with amines.

Our procedure was successfully applied to a broad spectrum of readily available carboxylic acids and amines affording, in short times and after a simple work up, the corresponding amides in high purity and yields.

By using *N*-Boc-l-alanine and its enantiomer *N*-Boc-d-alanine as chiral carboxylic acids, and aniline as amine component, highly enantiomerically enriched anilides were synthesized, demonstrating that the developed procedure does not generate any significant loss of the optical integrity of the precursors.

The current investigation showed also that this approach works well also with carboxylic acids bearing acid-sensitive groups.

## Experimental

### General experimental details

All reagents were purchased commercially without further purification. Solvents were purified according to well-known laboratory methods and freshly distilled prior to use. Reaction were carried out in a tightly sealed screw-capped vial. Reactions were magnetically stirred and monitored by thin layer chromatography using Merck-Kieselgel 60 F254 plates. ^1^H and ^13^C NMR spectra were recorded on a Bruker Avance 300 instrument at 300 MHz and 75 MHz, respectively. Spectroscopic analysis was performed at 293 K on diluted solutions of each compound by using CDCl_3_ and DMSO-d_6_ as the solvents. Chemical shifts (δ) are reported in ppm. Coupling constants (J) are reported in Hertz (Hz) (Additional file 1). GC–MS analyses were performed with a DB-35MS (20 m × 0.18 mm, 35% Phenyl 65% dimethylpolysiloxane) capillary column. The mass detector was operated in the electron impact ionization mode (EI/MS) with an electron energy of 70 eV. The injection port was heated to 250 °C. The oven temperature program was initially set at 70 °C with a hold of 2 min and ramped to 280 °C at 20 °C/min with a hold of 10 min. Chiral GC–MS analyses of enantiomeric compounds 27–28 were performed by using a 25 m × 0.25 mm, Diethyl tertbutyldimethylisilyl-β-cyclodextrine chiral capillary column. The injection port was heated to 250 °C. The oven temperature program was initially set at 50 °C, with a hold of 2 min, ramped to 250 °C at 0.5 °C/min with a hold of 5 min.

### General procedure for the synthesis of amides **1**–**28**

TiCl_4_ (3 mmol) and the amine (1 mmol) were added to a solution of carboxylic acid (1 mmol) in pyridine (10 mL). The tightly sealed screw-capped vial containing the reaction mixture was then heated at 85 °C. After magnetic stirring for about 2 h, TLC analysis (chloroform/methanol 90:10 v/v) of the reaction mixture showed complete conversion of the carboxylic acid precursor. The reaction mixture was then cooled, and after removing pyridine by co-evaporation with toluene, was treated with an aqueous 1 N HCl solution (10 mL) and extracted with methylene chloride (3 × 10 mL). The combined organic extracts were washed with a saturated aqueous solution of sodium bicarbonate (3 × 10 mL), dried (Na_2_SO_4_), and evaporated to dryness under reduced pressure to afford the corresponding amides **1**–**28** with yields ranging from 56 to 98%.

#### N-phenylbenzamide (**1**)

Solid (98%); mp = 163–165 °C; Rf = 0.70; ^1^H NMR (300 MHz, DMSO-d_6_) δ: 10.23 (s, 1H), 7.99–7.95 (m, 2H), 7.81–7.78 (m, 2H), 7.56–7.52 (m, 3H), 7.38–7.32 (m, 2H), 7.12–7.07 (m, 1H); ^13^C-NMR (75 MHz, DMSO-d_6_) δ: 166.0, 139.7, 135.5, 131.9, 129.0, 128.8, 128.1, 124.1, 120.9; GC/MS (EI) *m/z* (% rel.): 197 [M^+∙^] (46), 105 (100), 77 (46); 65 (4), 51 (9).

#### 4-nitro-N-phenylbenzamide (**2**)

Solid (98%); mp = 218–220 °C; Rf = 0.65; ^1^H NMR (300 MHz, DMSO-d_6_) δ: 10.53 (s, 1H), 8.35 (d, J = 9.0 Hz, 2H), 8.18 (d, J = 9.0 Hz,2H), 7.78 (d, J = 7.5 Hz, 2H), 7.46–7.27 (m, 2H), 7.13 (t, J = 7.4 Hz, 1H); ^13^C-NMR (75 MHz, DMSO-d_6_) δ: 164.3, 149.6, 141.1, 139.2, 129.7, 129.1, 124.6, 124.0, 121.0; GC/MS (EI) *m/z* (% rel.): 242 [M^+∙^] (75), 150 (100), 120 (21), 104 (32), 92 (20), 76 (25).

#### 4-methoxy-N-phenylbenzamide (**3**)

Solid (95%); mp = 177–179 °C; Rf = 0.69; ^1^H NMR (300 MHz, DMSO-d_6_) δ: 10.06 (s, 1H), 7.99–7.88 (m, 2H), 7.80–7.74 (m, 2H), 7.39–7.24 (m, 2H), 7.13–6.99 (m, 3H), 3.83 (s, 3H); ^13^C-NMR (75 MHz, DMSO-d_6_) δ: 165.4, 162.4, 139.9, 130.0, 129.0, 127.5, 123.9, 120.9, 114.1, 55.9; GC/MS (EI) *m/z* (% rel.): 227 [M^+∙^] (21), 135 (100), 107 (6), 92 (11), 77(12).

#### 4-chloro-N-phenylbenzamide (**4**)

Solid (95%); mp = 205–207 °C; Rf = 0.78; ^1^H NMR (300 MHz, DMSO-d_6_) δ: 10.28 (s, 1H), 8.06–7.88 (m, 2H), 7.82–7.71 (m, 2H), 7.64–7.53 m, 2H), 7.40–7.28 (m, 2H), 7.10 (t, J = 7.4 Hz, 1H); ^13^C-NMR (75 MHz, DMSO-d_6_) δ: 164.9, 139.5, 136.9, 134.1, 130.1, 129.1, 128.9, 124.3, 120.9; GC/MS (EI) *m/z* (% rel.): 231 [M^+∙^] (31), 139 (100), 111 (31), 75 (12).

#### N,2-diphenylacetamide (**5**)

Solid (95%); mp = 105–107 °C; Rf = 0.73; ^1^H NMR (300 MHz, CDCl_3_) δ: 7.63 (s_broad_, 1H), 7.49–7.42 (m, 2H), 7.39–7.21 (m, 7H), 7.09 (t, J = 7.4 Hz, 1H), 3.70 (s, 2H); ^13^C-NMR (75 MHz, CDCl_3_) δ: 169.4, 137.8, 134.6, 129.5, 129.1, 128.9, 127.6, 124.4, 120.0, 44.7; GC/MS (EI) *m/z* (% rel.): 211 [M^+∙^] (49), 119 (11), 91(68), 93 (100), 77 (11), 65 (21).

#### N-phenylcinnamamide (**6**)

Solid (91%); mp = 155–157 °C; Rf = 0.72; ^1^H NMR (300 MHz, CDCl_3_) δ: 8.09 (s, 1H), 7.75 (d, J = 15.6 Hz, 1H), 7.69–7.59 (m, 2H), 7.52–7.39 (m, 2H), 7.38–7.23 (m, 5H), 7.12 (t, J = 7.4 Hz, 1H), 6.66 (d, J = 15.6 Hz, 1H).; ^13^C-NMR (75 MHz, CDCl_3_) δ: 164.7, 142.2, 138.2, 134.6, 129.9, 129.1, 128.8, 128.0, 124.5, 121.2, 120.4; GC/MS (EI) *m/z* (% rel.): 223 [M^+∙^] (25), 131 (100) 103 (36), 93 (19), 77 (24).

#### N-phenylpalmitamide (**7**)

Solid (88%), mp = 88–90 °C; Rf = 0.81; ^1^H NMR (300 MHz, CDCl_3_) δ: 7.53 (d, J = 8.3 Hz, 2H), 7.40 (s, 1H), 7.36–7.22 (m, 2H), 7.11 (m, 1H), 2.42–2.29 (m, 2H), 1.80–1.60 (m, 2H), 1.41–1.13 (m, 24H,), 0.89 (t, J = 6.7 Hz, 3H); ^13^C-NMR (75 MHz, CDCl_3_) δ: 171.7, 137.9, 129.0, 124.2, 119.8, 37.8, 31.9, 29.7, 29.6, 29.5, 29.4, 29.3, 25.7, 22.7, 14.2; GC/MS (EI) *m/z* (% rel.): 331 [M^+∙^] (2), 135 (19), 93 (100), 77 (2).

#### N-(2-fluorophenyl)-2-phenylacetamide (**8**)

Solid (72%), mp = 100–102 °C; Rf = 0.85; ^1^H NMR (300 MHz, CDCl_3_) δ: 8.29 (t, J = 8.0 Hz, 1H), 7.53–7.30 (m, 6H), 7.16–7.06 (m, 1H), 7.06–6.95 (m, 2H), 3.78 (s, 2H); ^13^C-NMR (75 MHz, CDCl_3_) δ: 169.2, 152,1 (d, J = 242.5 Hz), 134.1, 129.5, 129.3, 127.8, 126.2 (d, J = 10.3 Hz), 124.54 (d, J = 3.8 Hz), 124.51 (d, J = 7.6 Hz), 121.7, 114.7 (d, J = 19.4 Hz), 44.8; GC/MS (EI) *m/z* (% rel.): 229 [M^+∙^] (25), 118 (24), 111 (100), 91 (76), 65 (13).

#### 2-phenyl-N-(p-tolyl)acetamide (**9**)

Solid (98%), mp = 108–110 °C; Rf = 0.73; ^1^H NMR (300 MHz, CDCl_3_) δ: 7.52 (s_broad_, 1H), 7.43–7.28 (m, 7H), 7.07 (d, J = 8.3 Hz, 2H), 3.69 (s, 2H), 2.29 (s, 3H); ^13^C-NMR (75 MHz, CDCl_3_) δ: 169.3, 135.2, 134.7, 134.0, 129.5, 129.4, 129.1, 127.5, 120.1, 44.6, 20.9; GC/MS (EI) *m/z* (% rel.): 225 [M^+∙^] (49), 133 (12), 107 (100), 91 (46), 77 (9), 65 (12).

#### N-propylbenzamide (**10**)

Solid (91%); mp = 83–85 °C; Rf = 0.64; ^1^H NMR (300 MHz, CDCl_3_) δ: 7.82–7.69 (m, 2H), 7.52–7.27 (m, 3H), 6.60 (s_broad_, 1H), 3.42–3.31 (m, 2H), 1.70–1.51 (m, 2H), 0.93 (t, J = 7.4 Hz, 3H); ^13^C-NMR (75 MHz, CDCl_3_) δ: 167.6, 134.8, 131.1, 128.4, 126.8, 41.7, 22.8, 11.3; GC/MS (EI) *m/z* (% rel.): 163 [M^+∙^] (28), 134 (7), 105 (100), 77 (30).

#### 4-nitro-N-propylbenzamide (**11**)

Solid (92%); mp = 102–104 °C; Rf = 0.50; ^1^H NMR (300 MHz, CDCl_3_) δ: δ: 8.21 (d, J = 8.8 Hz, 2H), 7.92 (d, J = 8.8 Hz, 2H), 6.74 (s_broad_, 1H), 3.44–3.34 (m, 2H), 1.74–1.53 (m, 2H), 0.95 (t, J = 7.4 Hz, 3H); ^13^C-NMR (75 MHz, CDCl_3_) δ: 165.6, 149.5, 140.5, 127.6, 123.5, 41.96, 22.6, 11.3; GC/MS (EI) *m/z* (% rel.): 208 [M^+∙^] (21), 193 (12), 179 (12), 150 (100), 104 (23), 76 (15).

#### 4-methoxy-N-propylbenzamide (**12**)

Solid (78%); mp = 58–60 °C; Rf = 0.54; ^1^H NMR (300 MHz, CDCl_3_) δ: 7.74 (d, J = 8.8 Hz, 2H,), 6.87 (d, J = 8.8 Hz, 2H), 6.45 (s_broad_, 1H), 3.81 (s, 3H), 3.42–3.28 (m, 2H), 1.70–150 (m, 2H), 0.94 (t, J = 7.4 Hz, 3H); ^13^C-NMR (75 MHz, CDCl_3_) δ: 167.1, 162.0, 128.7, 127.1, 113.6, 55.4, 41.7, 22.9, 11.5; GC/MS (EI) *m/z* (% rel.): 193 [M^+∙^] (19), 151 (10), 135 (100), 107 (5), 92 (10), 77 (12).

#### 4-chloro-N-propylbenzamide (**13**)

Solid (96%); mp = 96–98 °C; Rf = 0.58; ^1^H NMR (300 MHz, CDCl_3_) δ: 7.75–7.65 (m, 2H), 7.41–7.28 (m, 2H), 6.55 (s_broad_, 1H), 3.43–3.28 (m, 2H), 1.70–1.50 (m, 2H), 0.94 (t, J = 7.4 Hz, 3H); ^13^C-NMR (75 MHz, CDCl_3_) δ: 166.5, 137.4, 133.2, 128.6, 128.3, 41.8, 22.7, 11.3; GC/MS (EI) *m/z* (% rel.): 197 [M^+∙^] (25), 168 (6), 139 (100), 111 (23), 75 (11).

#### 2-phenyl-N-propylacetamide (**14**)

Solid (95%); mp = 66–69 °C; Rf = 0.68; ^1^H NMR (300 MHz, CDCl_3_) δ: 7.42–7.17 (m, 5H), 5.65 (s_broad_, 1H), 3.55 (s, 2H), 3.22–3.07 (m, 2H), 1.58–1.31 (m, 2H), 0.82 (t, J = 7.4 Hz, 3H); ^13^C-NMR (75 MHz, CDCl_3_) δ: 171.0, 135.1, 129.4, 129.0, 127.3, 43.8, 41.3, 22.7, 11.2; GC/MS (EI) *m/z* (% rel.): 177 [M^+∙^] (18), 92 (100), 91 (87), 86 (10), 65 (13), 43 (23).

#### N-propylcinnamamide (**15**)

Solid (97%); mp = 75–77 °C; Rf = 0.71; ^1^H NMR (300 MHz, CDCl_3_) δ: 7.62 (d, J = 15.6 Hz, 1H), 7.54–7.41 (m, 2H), 7.38–7.25 (m, 3H), 6.50 (d, J = 15.6 Hz, 1H), 6.34 (sbroad, 1H), 3.47–3.26 (m, 2H), 1.71–1.49 (m, 2H), 0.94 (t, J = 7.4 Hz, 3H); ^13^C-NMR (75 MHz, CDCl_3_) δ: 166.1, 140.5, 134.9, 128.7, 127.7, 127.7, 121.1, 41.4, 22.8, 11.4; GC/MS (EI) *m/z* (% rel.): 189 [M^+∙^] (27), 131 (100), 174 (5), 146 (24), 103 (40), 77 (24).

#### N-propylpalmitamide (**16**)

Solid (94%), mp = 74–76 °C; Rf = 0.81; ^1^H NMR (300 MHz, CDCl_3_) δ: 5.49 (s_broad_, 1H), 3.27–3.14 (m, 2H), 2.20–2.09 (m, 2H), 1.73–1.58 (m, 2H), 1.57–1.41 (m, 2H), 1.40–1.11 (m, 24H), 0.98–0.78 (m, 6H); ^13^C-NMR (75 MHz, CDCl_3_) δ: 173.1, 41.2, 37.0, 31.9, 29.7, 29.6, 29.5, 29.4, 29.3, 25.9, 22.9, 22.7, 14.1, 11.4; GC/MS (EI) *m/z* (% rel.): 297 [M^+∙^] (3), 268 (3), 239 (3), 114 (24), 101 (100), 43 (14).

#### N,N-diethylbenzamide (**17**)

Viscous oil (64%); Rf = 0.75; ^1^H NMR (300 MHz, CDCl_3_) δ: 7.41–7.10 (m, 5H), 3.43 (s_broad_, 2H), 3.14 (s_broad_, 2H), 1.27–0.89 (m, 6H); ^13^C-NMR (75 MHz, CDCl_3_) δ: 171.1, 137.1, 128.9, 128.2, 126.1, 43.1, 39.1, 14.0, 12.7; GC/MS (EI) *m/z* (% rel.): 177 [M^+∙^] (21), 162 (2), 148 (7), 176 (61), 105 (100), 77 (27).

#### N,N-diethyl-4-nitrobenzamide (**18**)

Solid (80%); mp = 54–56 °C; Rf = 0.73; ^1^H NMR (300 MHz, CDCl_3_) δ: 8.26 (d, J = 8.8 Hz, 2H), 7.53 (d, J = 8.8 Hz, 2H), 3.73–3.36 (m, 2H), 3.34–3.01 (m, 2H), 1.25 (t, J = 6.9 Hz, 3H), 1.11 (t, J = 6.9 Hz, 3H); ^13^C-NMR (75 MHz, CDCl_3_) δ: 168.9, 148.3, 143.4, 127.3, 123.9, 43.3, 39.5, 14.2, 12.8; GC/MS (EI) *m/z* (% rel.): 222 [M^+∙^] (17), 221 (42), 205 (7), 175 (4), 150 (100), 120 (13), 104 (25), 92 (8), 76 (14).

#### N,N-diethyl-4-methoxybenzamide (**19**)

Oil (56%); Rf = 0.65; ^1^H NMR (300 MHz, CDCl_3_) δ: 7.22 (d, J = 8.7 Hz, 2H), 6.77 (d, J = 8.7 Hz, 2H), 3.68 (s, 3H,), 3.29 (s_broad_, 4H), 1.05 (s_broad_, 6H); ^13^C-NMR (75 MHz, CDCl_3_) δ: 171.1, 160.1, 129.3, 128.0, 113.5, 55.1, 43.0, 39.7, 13.5; GC/MS (EI) *m/z* (% rel.): 207 [M^+∙^] (15), 206 (34), 135 (100), 107 (4), 92 (11), 77 (9).

#### 4-chloro-N,N-diethylbenzamide (**20**)

Oil (77%); Rf = 0.65; ^1^H NMR (300 MHz, CDCl_3_) δ: 7.41–7.24 (m, 4H, ArH), 3.50 (s_broad_, 2H), 3.22 (s_broad_, 2H), 1.31–0.98 (m, 6H); ^13^C-NMR (75 MHz, CDCl_3_) δ: 170.2, 135.6, 135.1, 128.7, 127.8, 43.1, 39.1, 14.1, 12.7; GC/MS (EI) *m/z* (% rel.): 211 [M^+∙^] (15), 210 (34), 139 (100), 111 (23), 75 (9).

#### N,N-diethyl-2-phenylacetamide (**21**)

Oil (85%); Rf = 0.73; ^1^H NMR (300 MHz, CDCl_3_) δ: 7.36–7.15 (m, 5H), 3.69 (s, 2H), 3.38 (q, J = 7.1 Hz, 2H), 3.28 (q, J = 7.1 Hz, 2H), 1.16–1.02 (m, 6H); ^13^C-NMR (75 MHz, CDCl_3_) δ: 170.2, 135.5, 128.7, 128.6, 126.6, 42.4, 40.9, 40.1, 14.2, 12.9; GC/MS (EI) *m/z* (% rel.): 191[M^+∙^] (38), 118 (3), 100 (100), 91 (46), 72 (50).

#### N,N-diethylcinnamamide (**22**)

Solid (87)  %); mp = 58-60 °C; Rf = 0.73; ^1^H NMR (300 MHz, CDCl3) δ: 7.55 (d, J = 15.4 Hz, 1H), 7.41–7.27 (m, 2H), 7.24–7.04 (m, 3H), 6.68 (d, J = 15.4 Hz, 1H), 3.42–3.15 (m, 4H), 1.16–0.92 (m, 6H); ^13^C-NMR (75 MHz, CDCl_3_) δ: 165.4, 141.9, 135.3, 129.2, 128.6, 127.5, 117.7, 42.1, 40.9, 14.9, 13.0; GC/MS (EI) *m/z* (% rel.): 203 [M^+∙^] (32), 188 (8), 131 (100), 126 (8), 103 (35), 77 (12).

#### N,N-diethylpalmitamide (**23**)

Oil (91)  %); Rf = 0.73; ^1^H NMR (300 MHz, CDCl_3_) δ: 3.42–3.21 (m, 4H), 2.35–2.19 (m, 2H), 1.71–1.51 (m, 2H), 1.39–0.99 (m, 30H), 0.87 (t, J = 6.7, 3H); ^13^C-NMR (75 MHz, CDCl_3_) δ: 172.4, 41.9, 40.0, 33.2, 31.9, 29.7, 29.6, 29.5, 29.4, 25.5, 22.7, 14.4, 14.1, 13.1; GC/MS (EI) *m/z* (% rel.): 311 [M^+∙^] (8), 128 (26), 115 (100), 100 (23).

#### N-phenylpivalamide (**24**)

Solid (90%); mp = 133–135 °C; Rf = 0.82; ^1^H NMR (300 MHz, CDCl_3_) δ: 7.57–7.50 (m, 2H), 7.43 (s_broad_, 1H), 7.36–7.25 (m, 2H), 7.14–7.04 (m, 1H), 1.32 (s, 9H); ^13^C-NMR (75 MHz, CDCl_3_) δ: 176.6, 138.0, 128.9, 124.2, 120.0, 39.6, 27.6; GC/MS (EI) *m/z* (% rel.): 177 [M^+∙^] (90), 120 (7), 93 (100), 77 (12), 57 (95).

#### N-propylpivalamide (**25**)

Oil (75%); Rf = 0.84; ^1^H NMR (300 MHz, CDCl_3_) δ: 5.73 (s, 1H), 3.23–3.11 (m, 2H), 1.58–1.40 (m, 2H), 1.16 (s, 9H), 0.88 (t, J = 7.4 Hz, 3H); ^13^C-NMR (75 MHz, CDCl_3_) δ: 178.4, 41.2, 38.6, 27.6, 22.8, 11.3; GC/MS (EI) *m/z* (% rel.): 143 [M^+∙^] (54), 128 (19), 114 (7), 100 (14), 86 (41), 85 (26), 57 (100), 43 (59).

#### N,N-diethylpivalamide (**26**)

oil (9%); Rf = 0.65; ^1^H NMR (300 MHz, CDCl_3_) δ: 3.48–3.32 (m, 4H), 1.29–1.18 (m, 15H); GC/MS (EI) *m/z* (% rel.): 157 [M^+∙^] (25), 142 (7); 100 (100), 72 (62), 57 (53).

#### (S)-2-(N-tert-Butoxycarbonylamino)-N-phenylpropanamide (**27**)

Oil (88%); Rf = 0.80; ^1^H NMR (300 MHz, CDCl_3_) δ: 8.86 (s_broad_, 1H), 7.58–7.45 (m, 2H), 7.36–7.19 (m, 2H), 7.13–7.01 (m, 1H), 5.50 (d, J = 7.5 Hz, 1H), 4.53–4.31 (m, 1H), 1.48–1.38 (m, 12H); ^13^C-NMR (75 MHz, CDCl_3_) δ: 171.2, 155.9, 137.9, 128.9, 124.2, 119.9, 80.4, 50.4, 28.3, 17.8; GC/MS (EI) *m/z* (% rel.): 264 [M^+∙^] (17), 208 (25), 191 (29), 144 (18), 120 (28), 93 (100), 77 (25), 57 (87).

#### (R)-2-(N-tert-Butoxycarbonylamino)-N-phenylpropanamide (**28**)

Oil (87%); Rf = 0.80; ^1^H NMR (300 MHz, CDCl_3_) δ: 8.69 (s_broad_, 1H), 7.61–7.42 (m, 2H), 7.33–7.21 (m, 2H), 7.11–7.02 (m, 1H), 5.31 (d, J = 8.0 Hz, 1H), 4.49–4.27 (m, 1H), 1.51–1.38 (m, 12H); ^13^C-NMR (75 MHz, CDCl_3_) δ: 171.1, 156.1, 137.8, 128.9, 124.2, 119.9, 80.7, 51.7, 28.3, 17.5; GC/MS (EI) *m/z* (% rel.): 264 [M^+∙^] (17), 208 (24), 191 (28), 144 (18), 120 (28), 93 (100), (77 (25), 57 (92).

## Additional files

**Additional file 1.** Supporting informations.
