# Supplementary material for: Formation of amides: one-pot condensation of carboxylic acids and amines mediated by TiCl4
Source: Chem Cent J. 2017 Sep 15;11:87. doi: 10.1186/s13065-017-0318-9 (PMC5602818; doi:10.1186/s13065-017-0318-9)

Supporting Informations for

Formation of amides: One-pot condensation of carboxylic acids and amines mediated by $TiCl_4$

Antonella Leggio,* Jessica Bagalà, Emilia Lucia Belsito, Alessandra Comandè, Marianna Greco and Angelo Liguori*

Dipartimento di Farmacia e Scienze della Salute e della Nutrizione, Università della Calabria, Edificio Polifunzionale, I-87036 Arcavacata di Rende- Italy

**Corresponding Authors*

Angelo Liguori E-mail: angelo.liguori@unical.it, tel: +39-0984 493205

Antonella Leggio E-mail: antonella.leggio@unical.it, tel: +39-0984 493199

Jessica Bagalà E-Mail: jessicabagala91@gmail.com

Emilia Lucia Belsito E-Mail: emilialucia.belsito@unical.it

Alessandra Comandè E-mail: alessandracomande@outlook.it

Marianna Greco E-mail: mariannagreco.89@gmail.com

Content:

1. ^{1}H NMR and ^{13}C NMR spectra of compounds **1-25** S1-25
2. ^{1}H NMR spectrum of compound **26** S26
3. ^{1}H NMR and ^{13}C NMR spectra of compounds **27-28** S27-28

***N*-phenylbenzamide (1)**

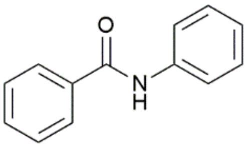

^{1}H NMR

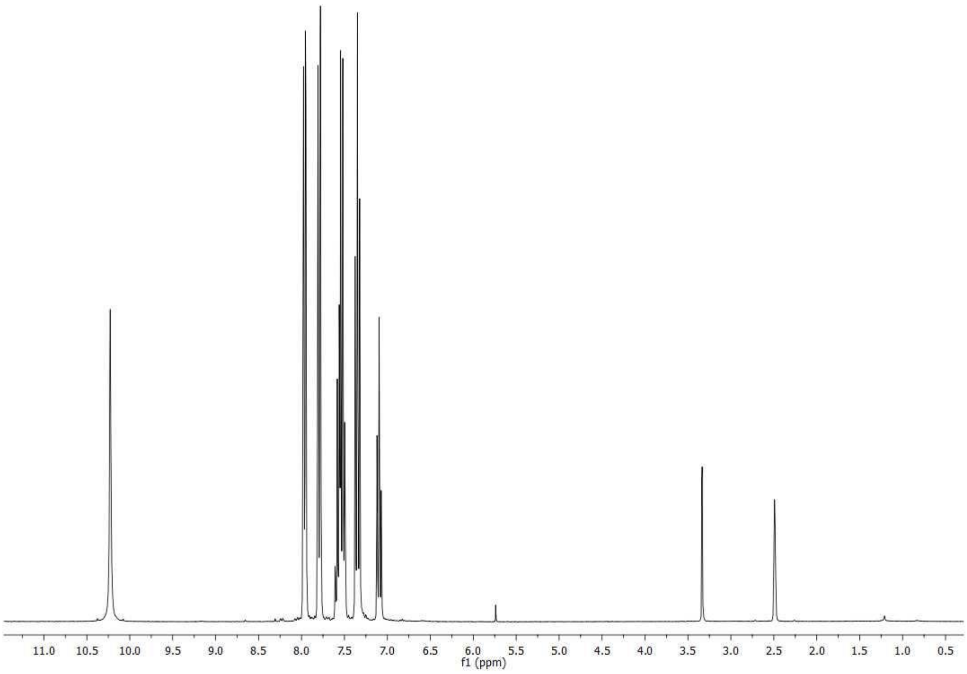

^{13}C NMR

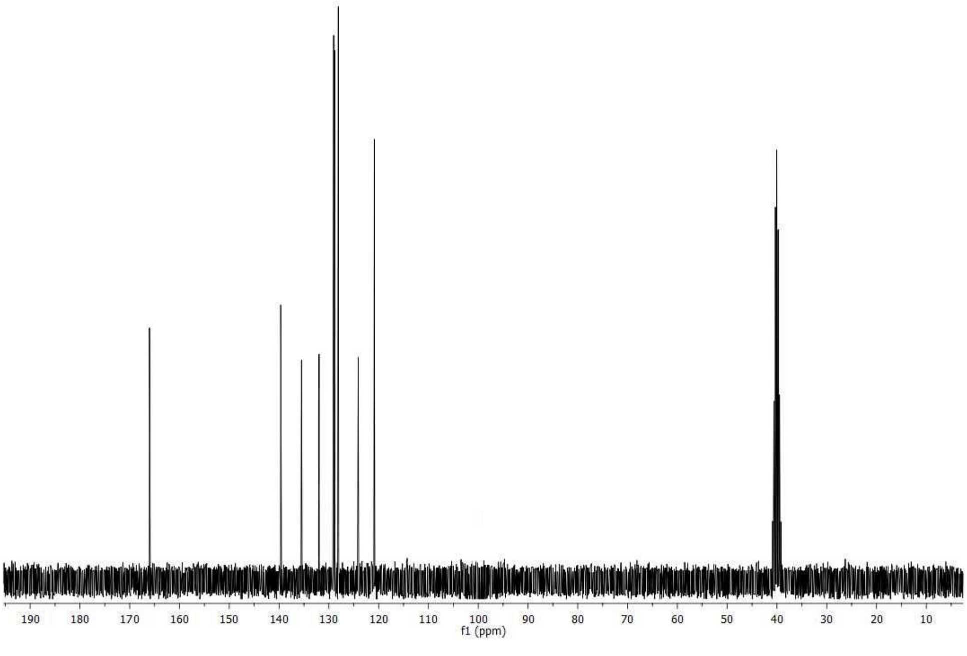

4-nitro-*N*-phenylbenzamide (2)

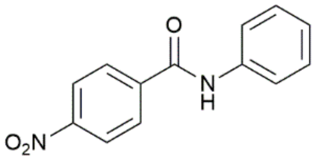

^{1}H NMR

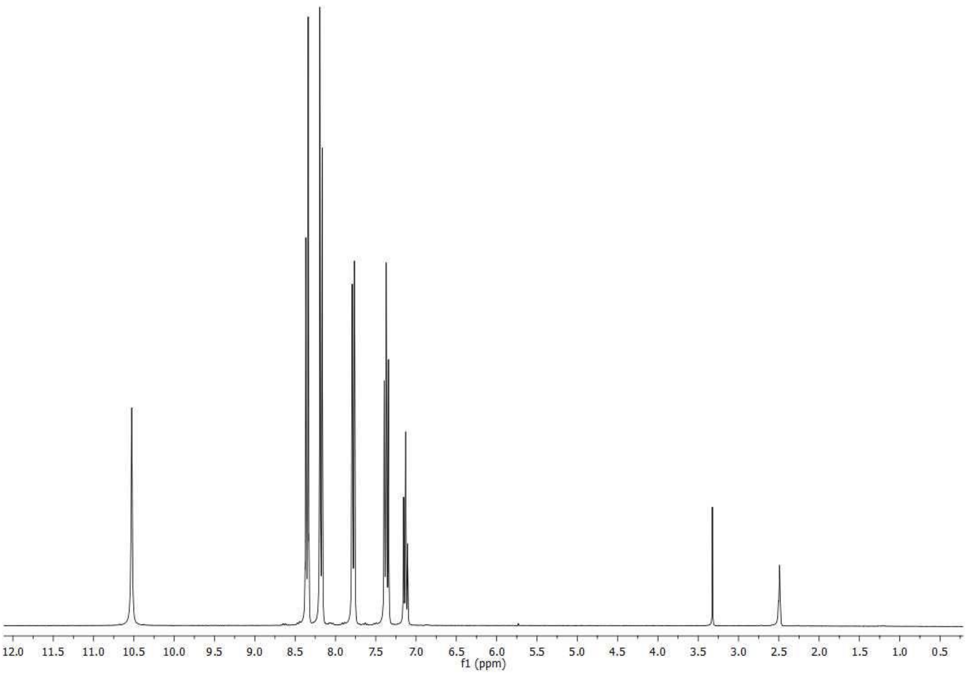

^{13}C NMR

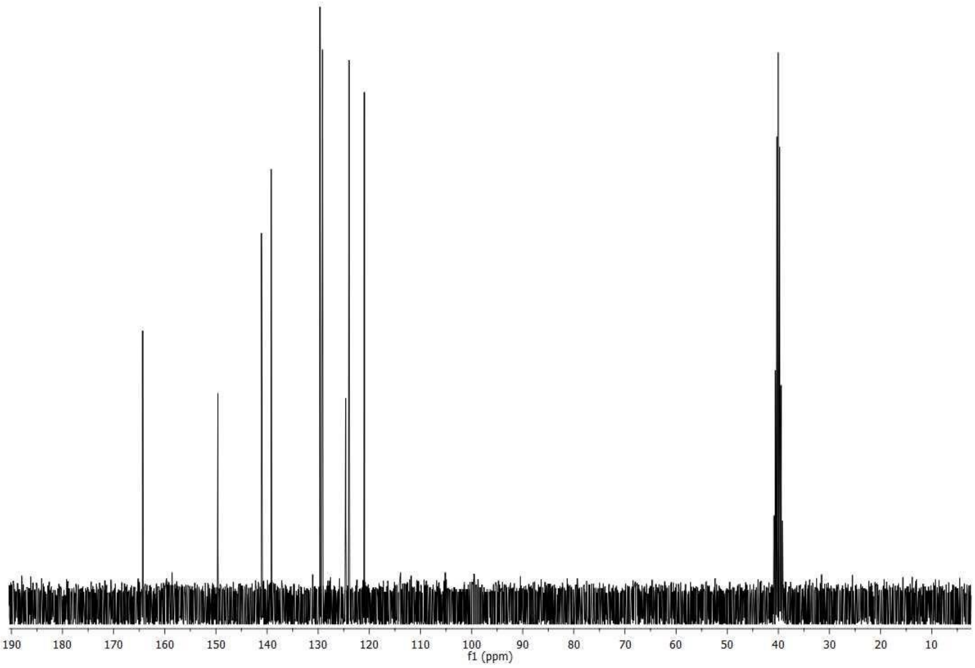

4-methoxy-*N*-phenylbenzamide (3)

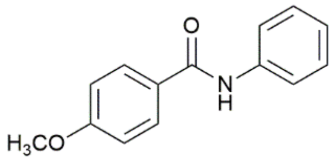

^{1}H NMR

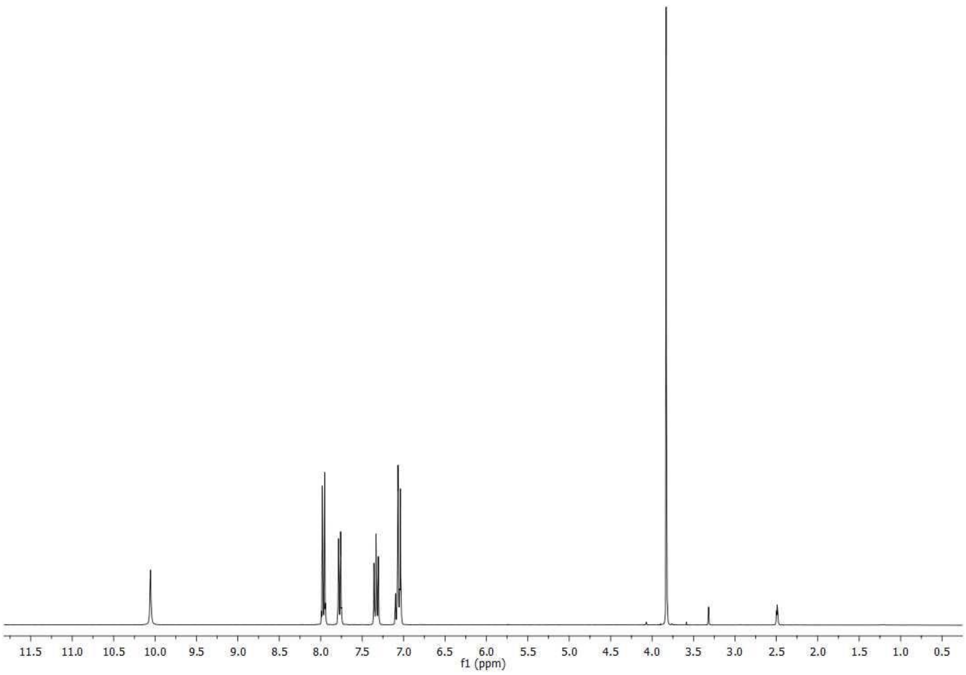

^{13}C NMR

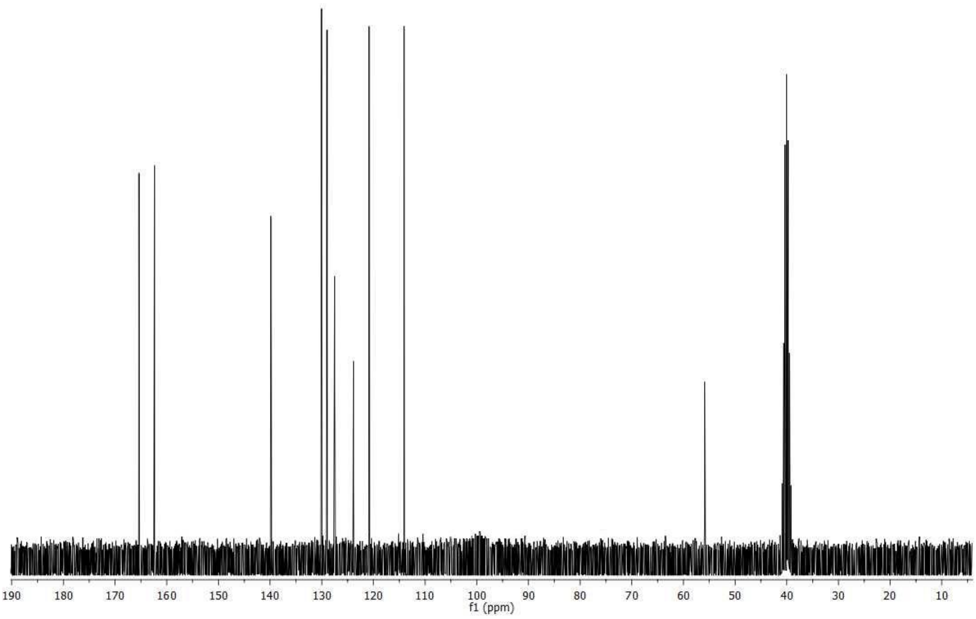

4-chloro-*N*-phenylbenzamide (4)

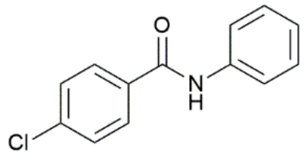

^{1}H NMR

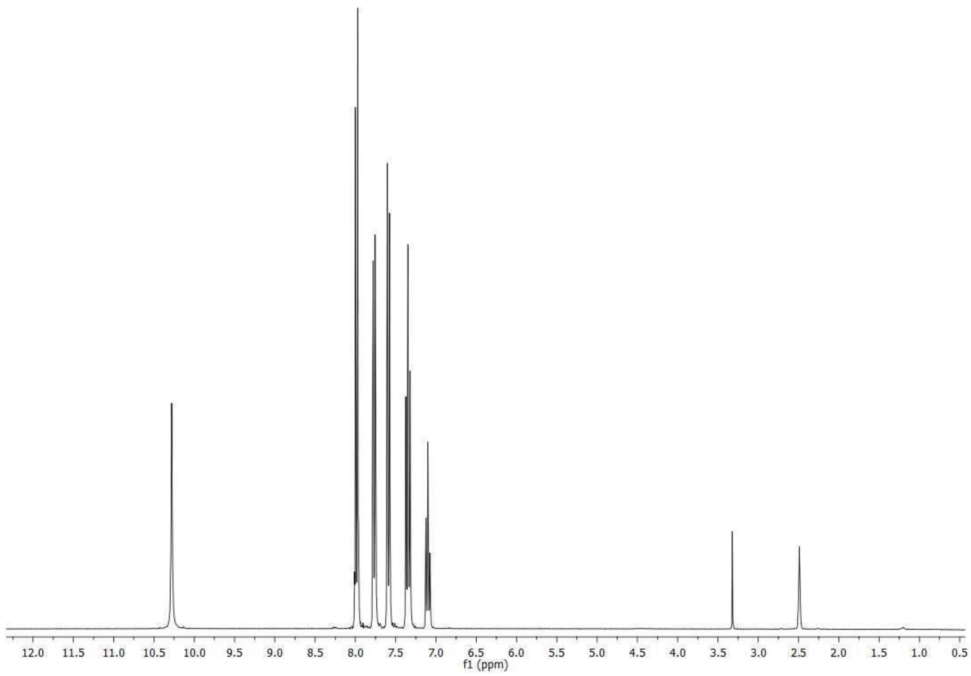

^{13}C NMR

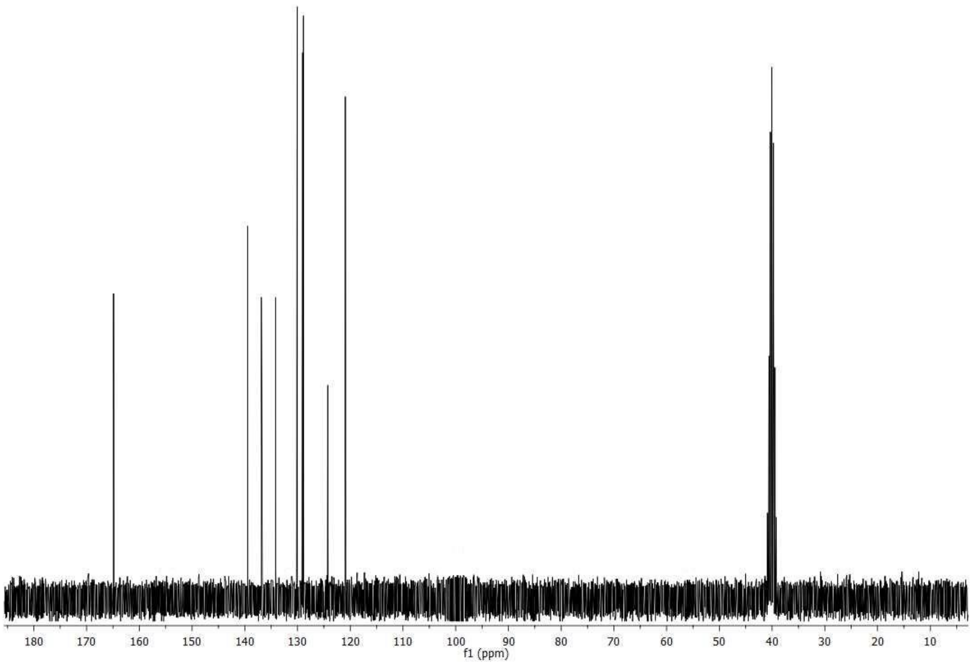

***N*,2-diphenylacetamide (5)**

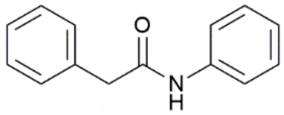

^{1}H NMR

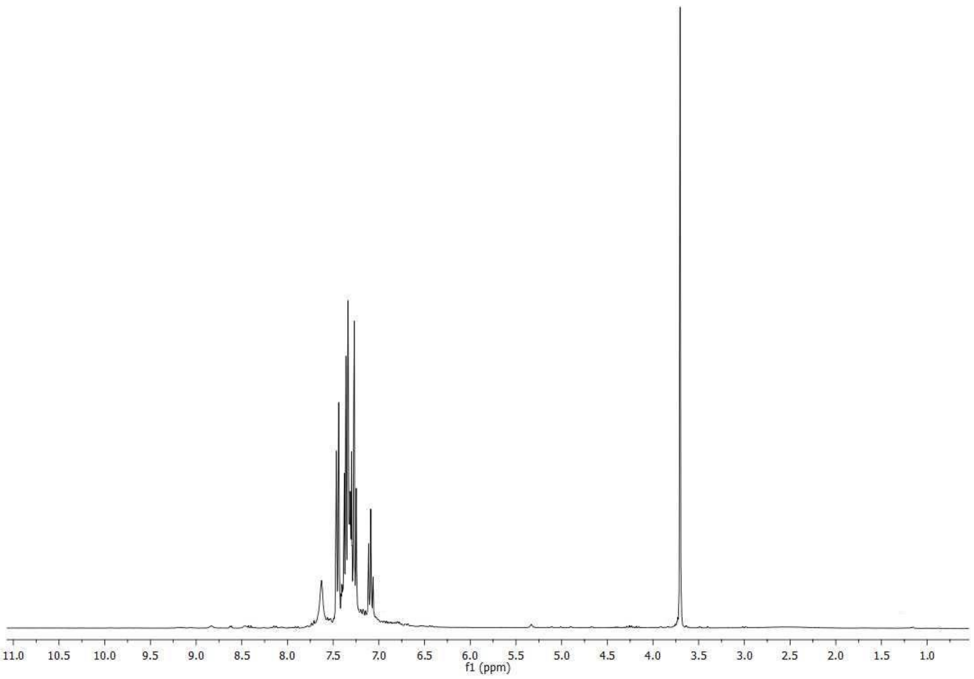

^{13}C NMR

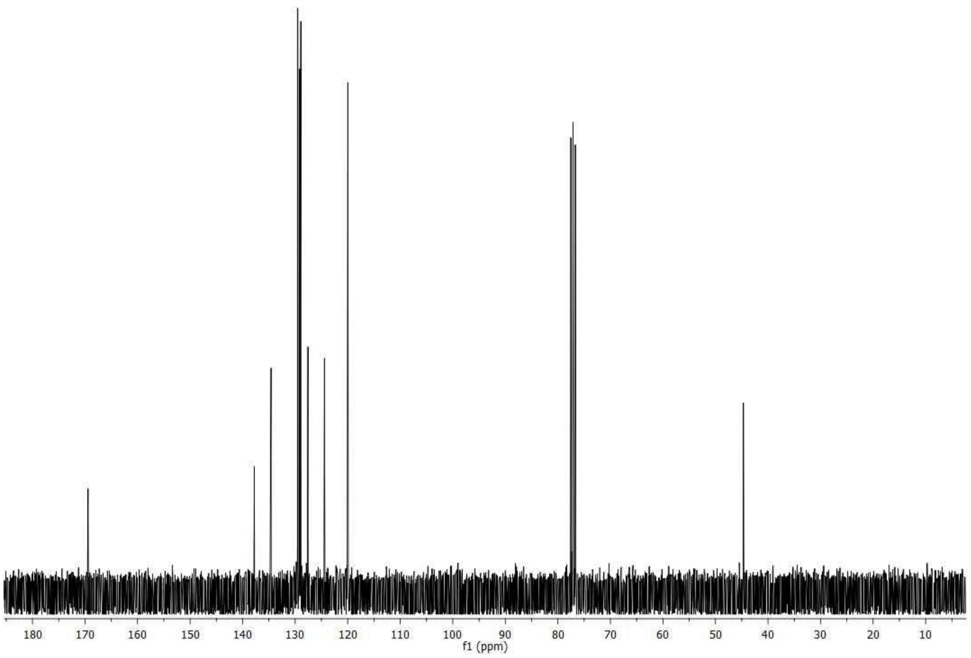

***N*-phenylcinnamamide (6)**

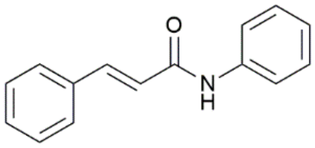

^{1}H NMR

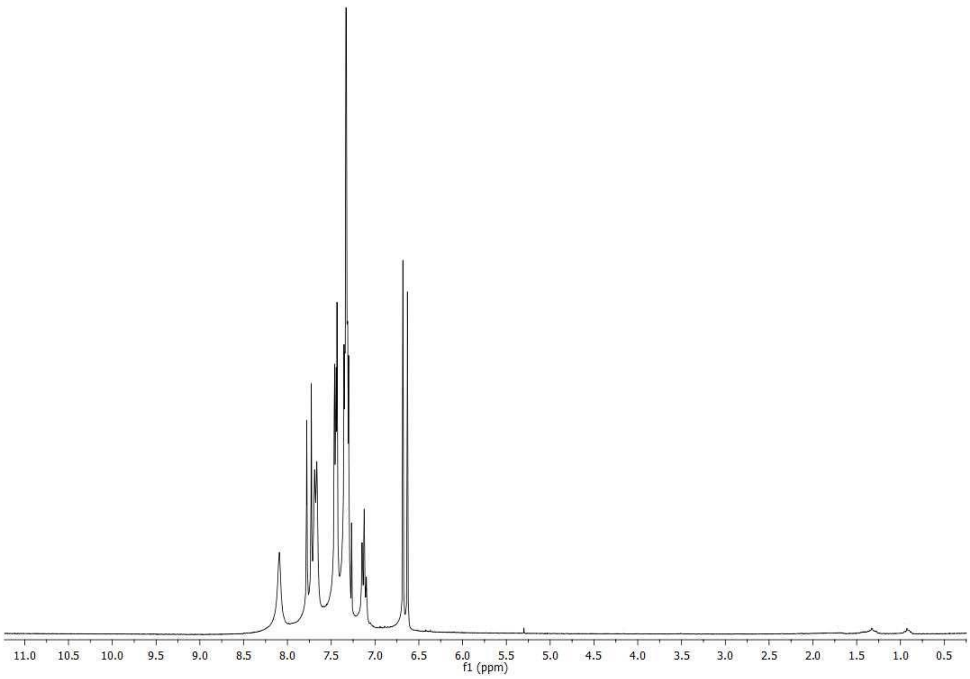

^{13}C NMR

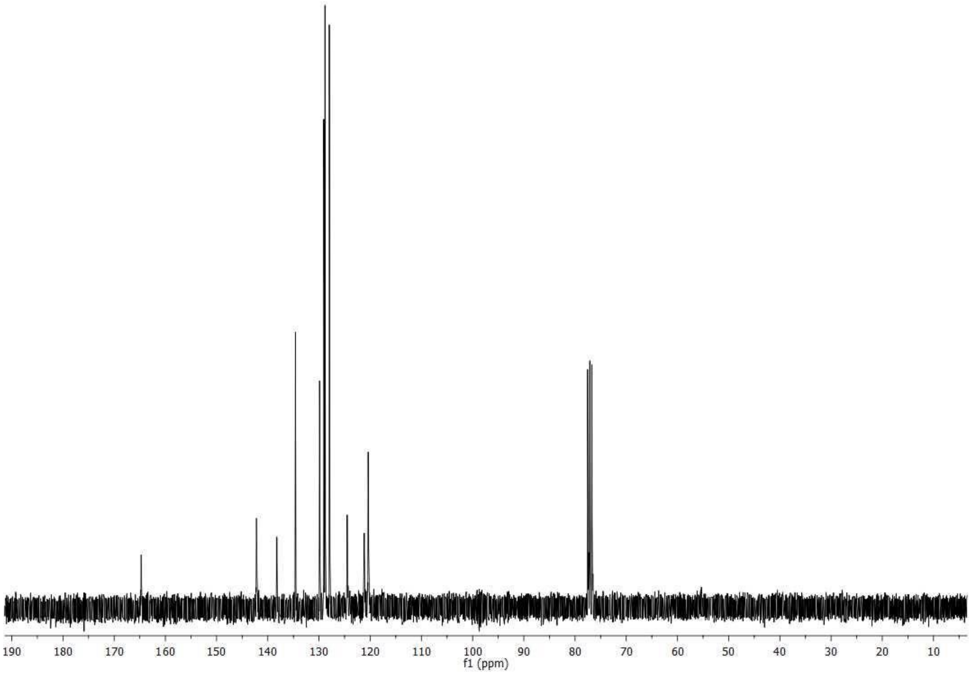

***N*-phenylpalmitamide (7)**

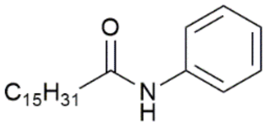

^{1}H NMR

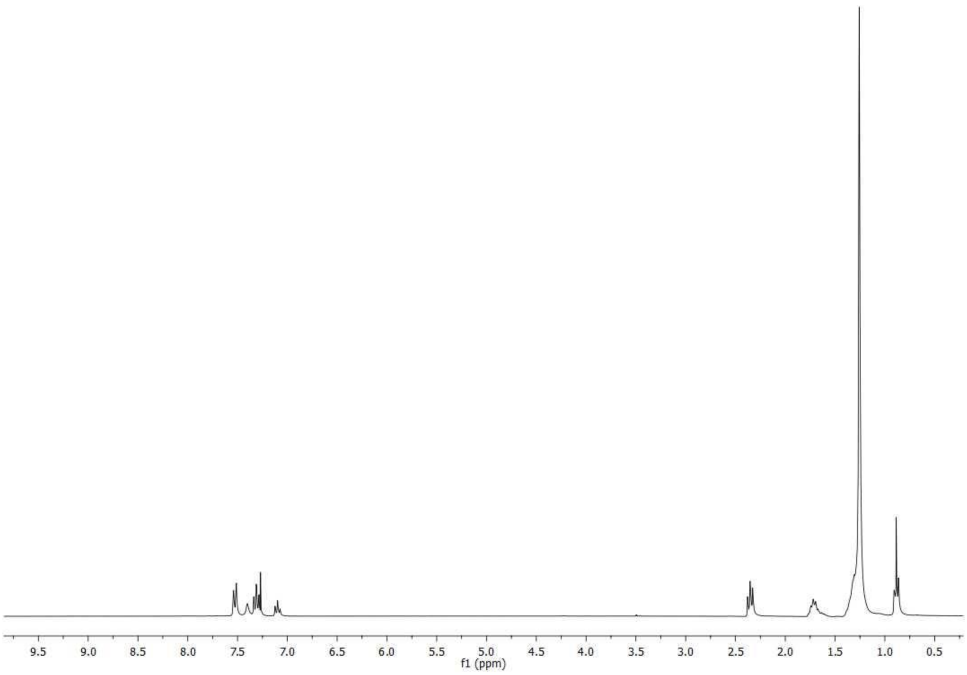

^{13}C NMR

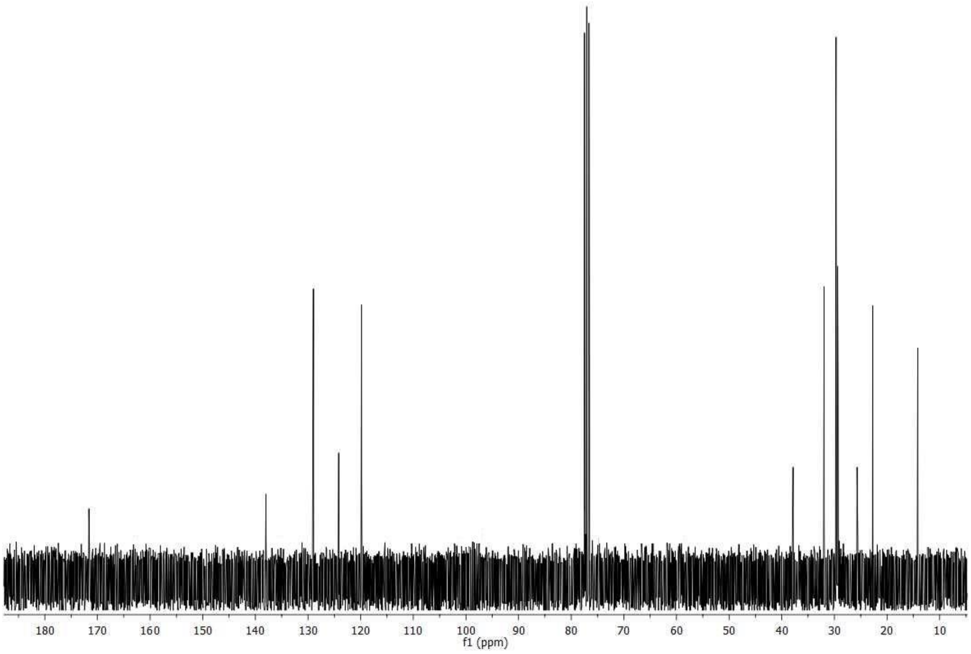

***N*-(2-fluorophenyl)-2-phenylacetamide (8)**

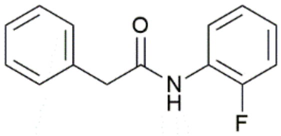

^{1}H NMR

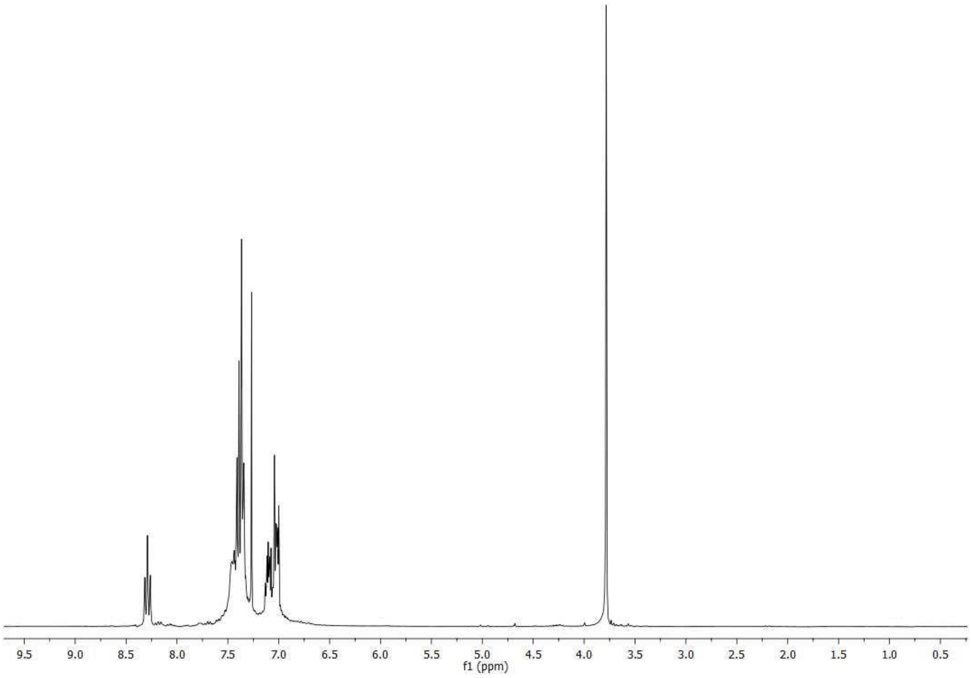

^{13}C NMR

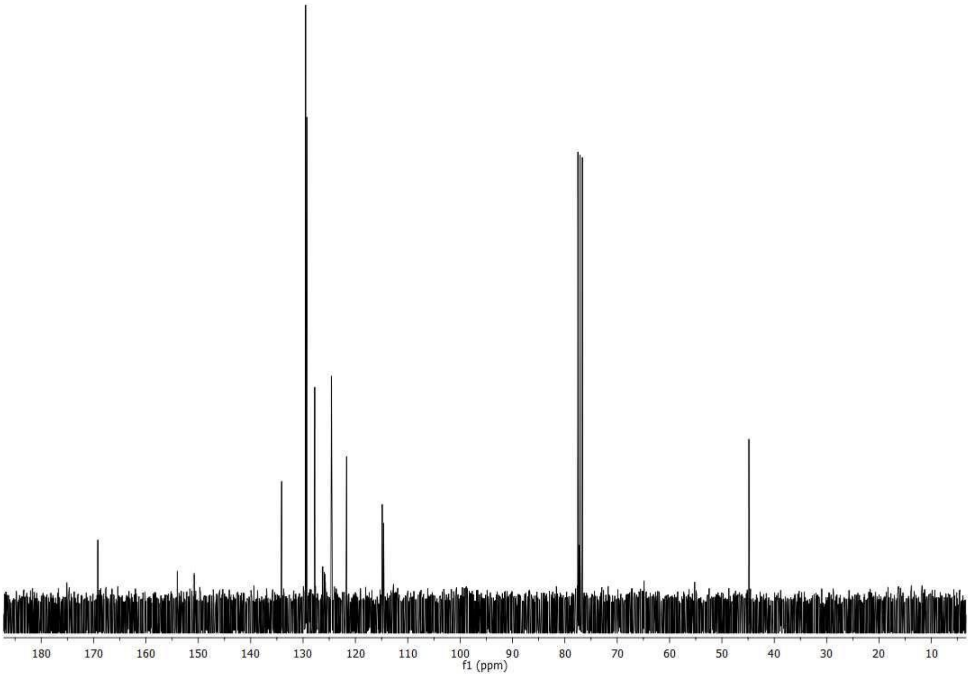

2-phenyl-*N*-(*p*-tolyl)acetamide (9)

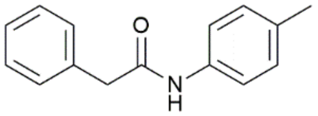

^{1}H NMR

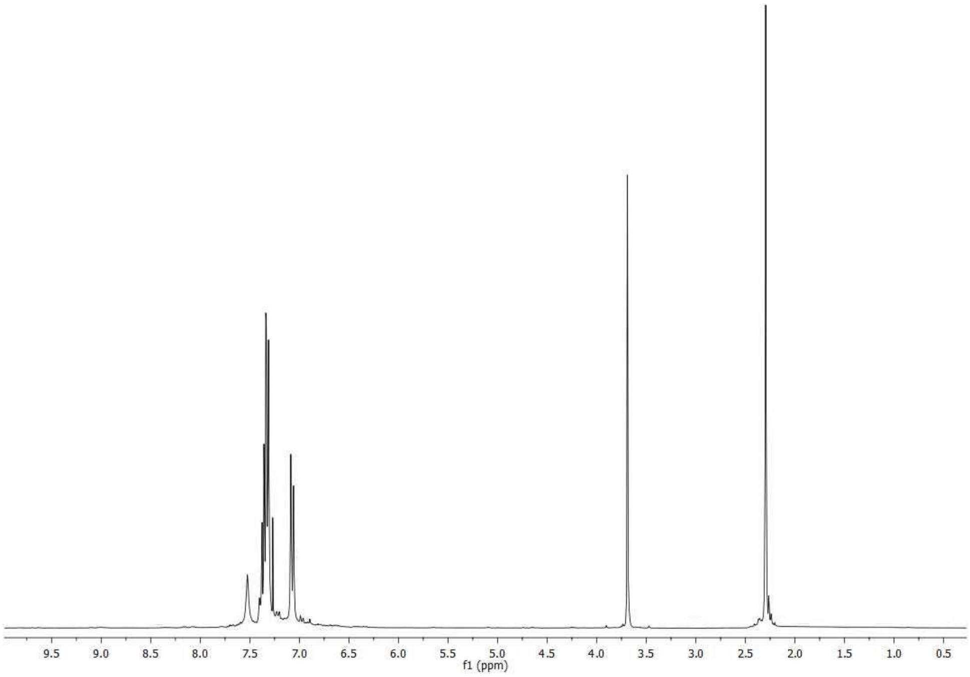

^{13}C NMR

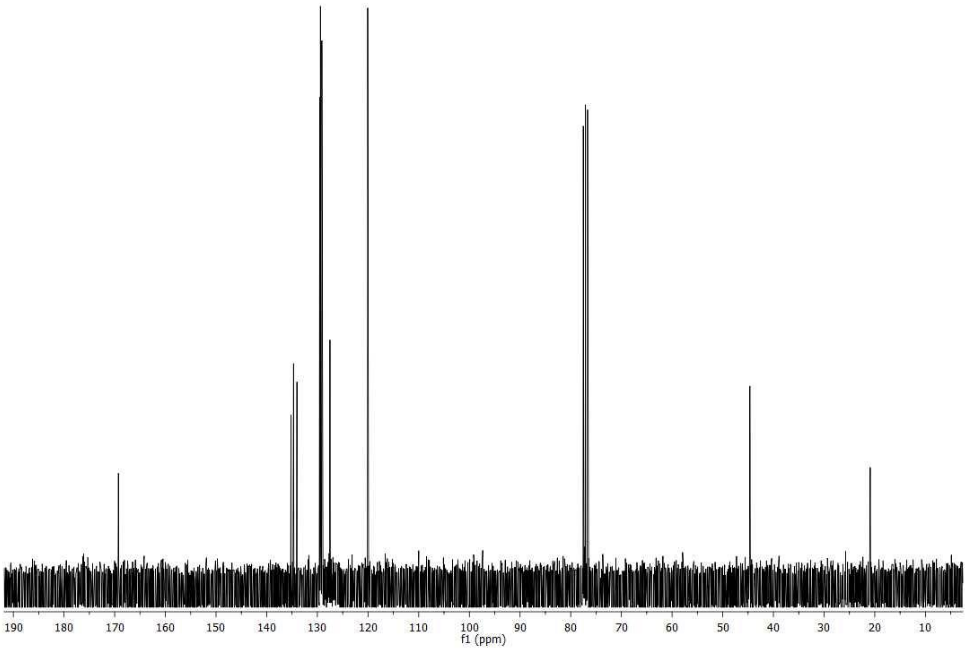

***N*-propylbenzamide (10)**

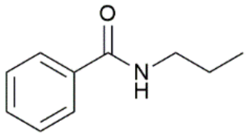

^{1}H NMR

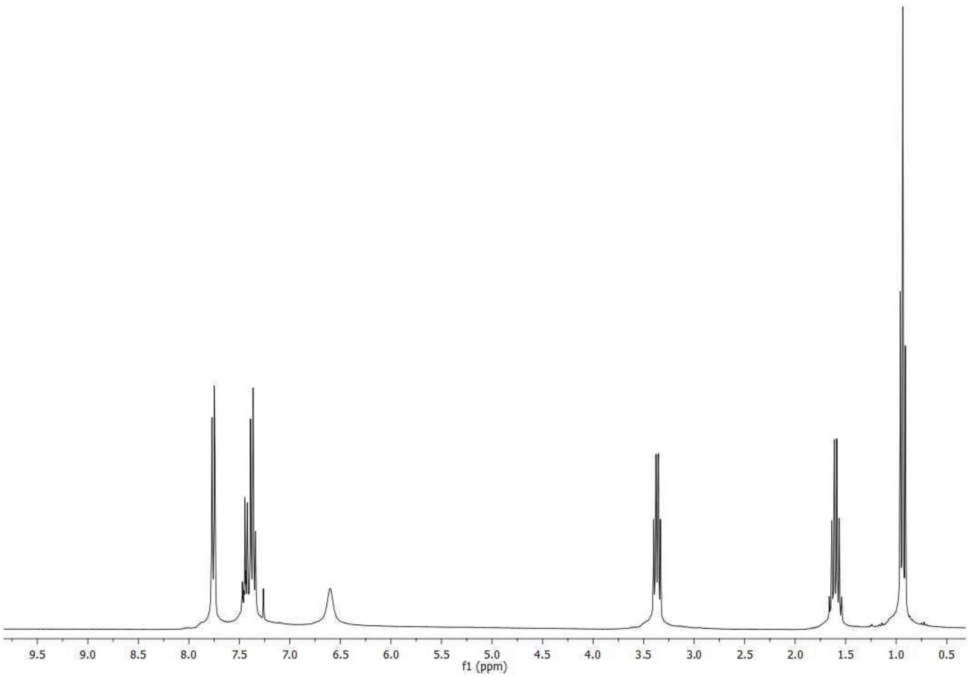

^{13}C NMR

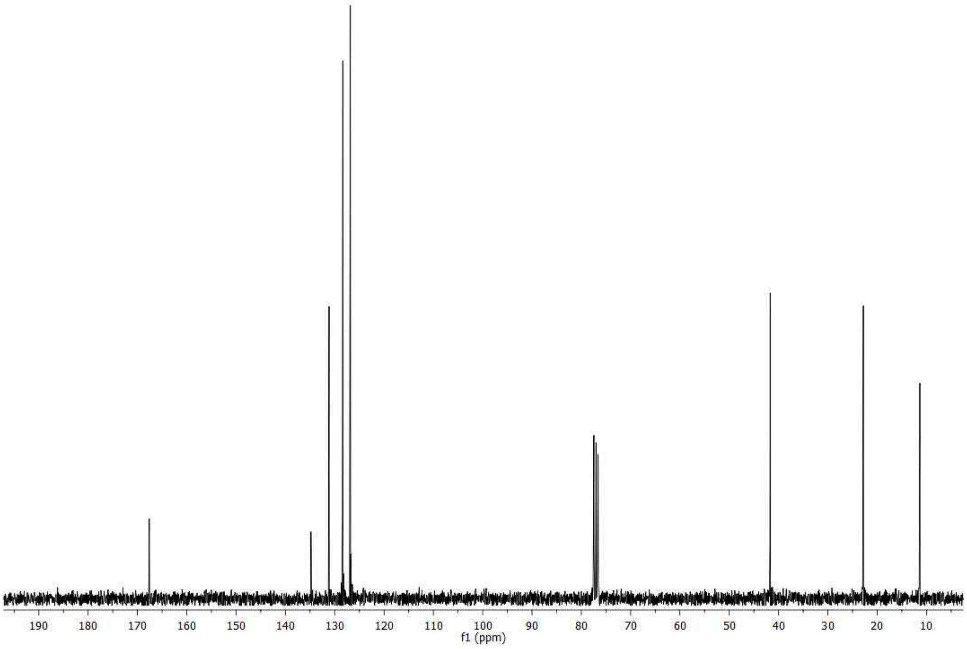

4-nitro-*N*-propylbenzamide (11)

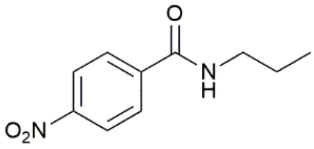

^{1}H NMR

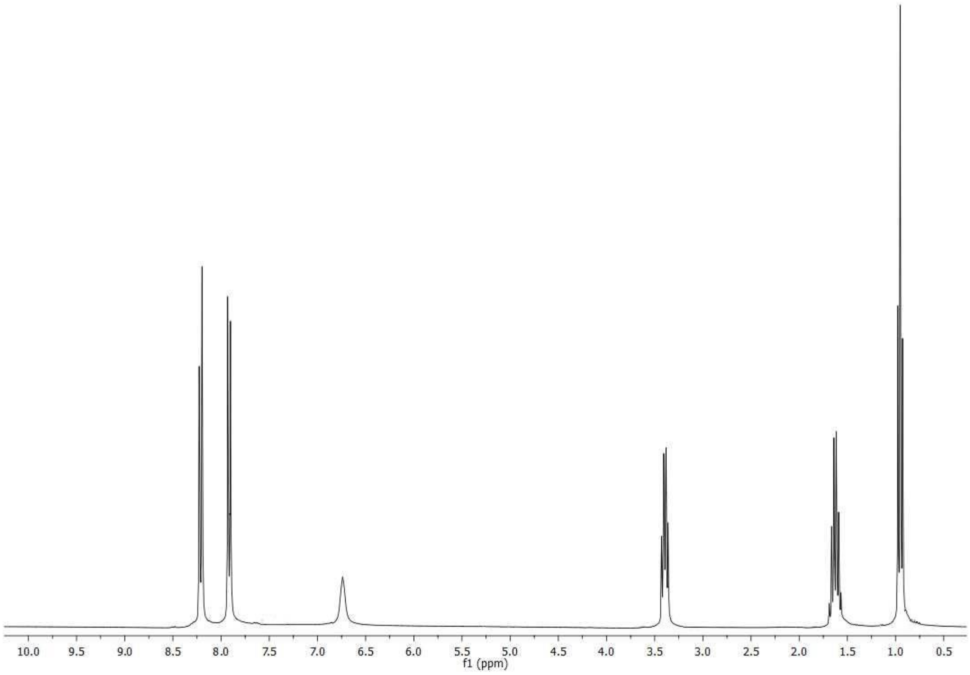

^{13}C NMR

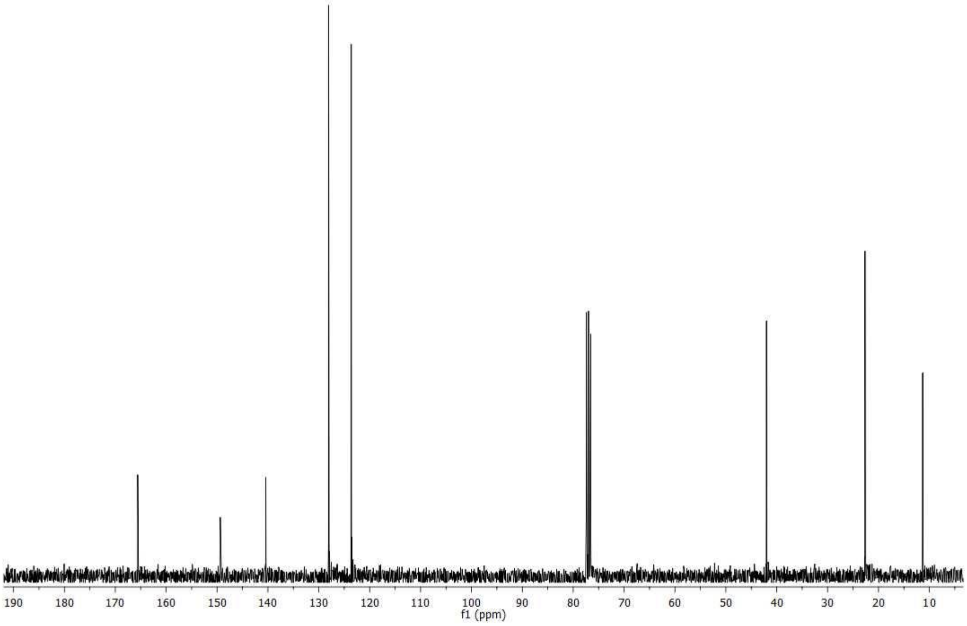

4-methoxy-*N*-propylbenzamide (12)

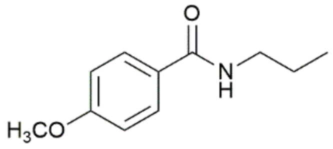

^{1}H NMR

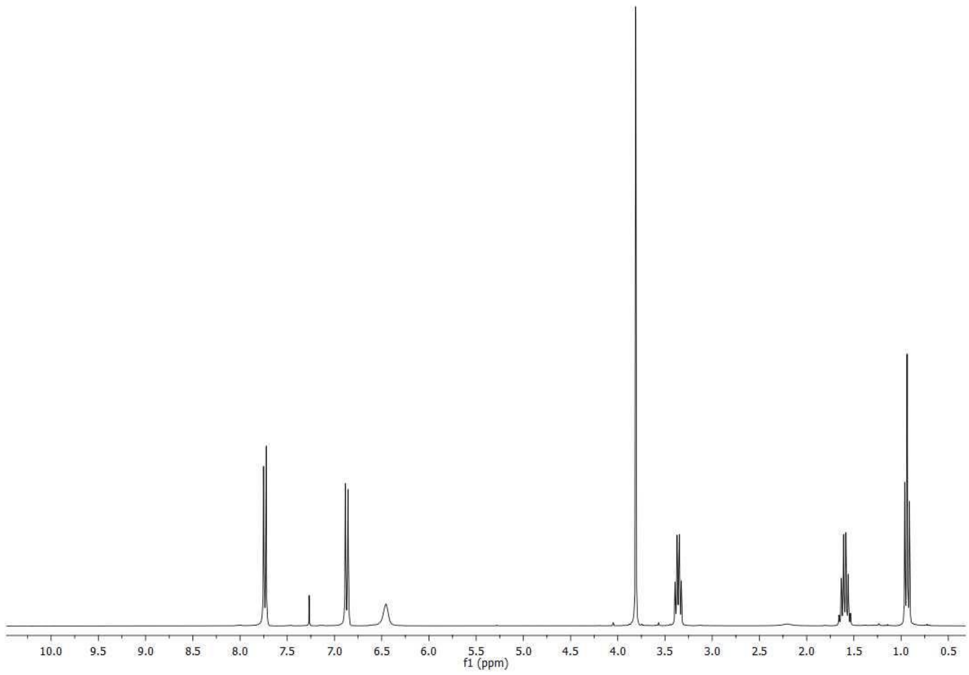

^{13}C NMR

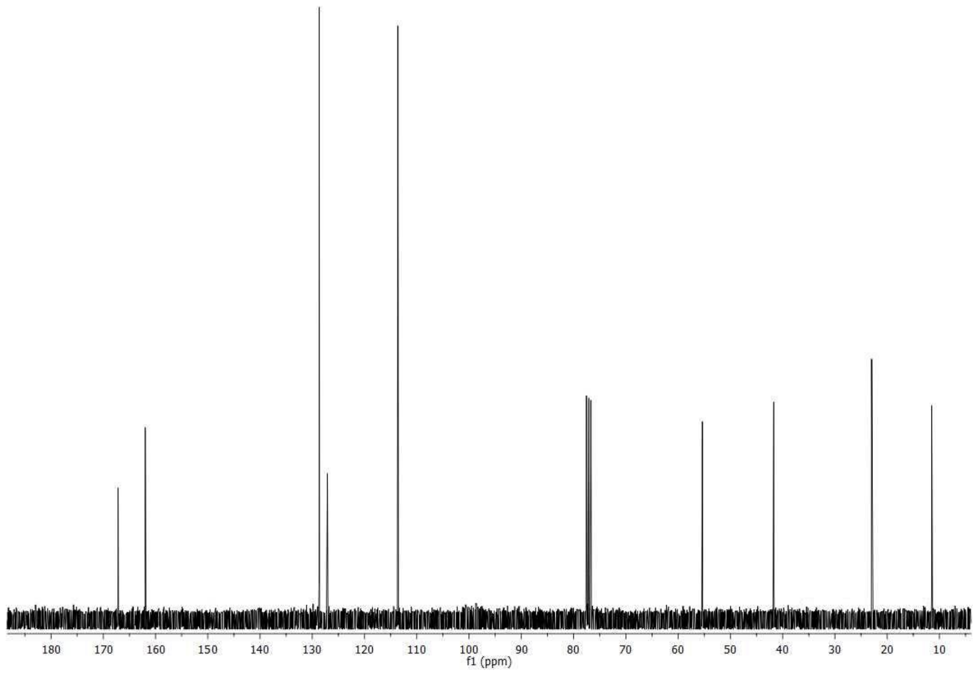

4-chloro-*N*-propylbenzamide (13)

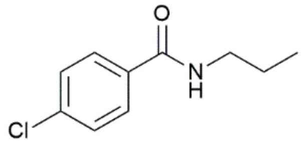

^{1}H NMR

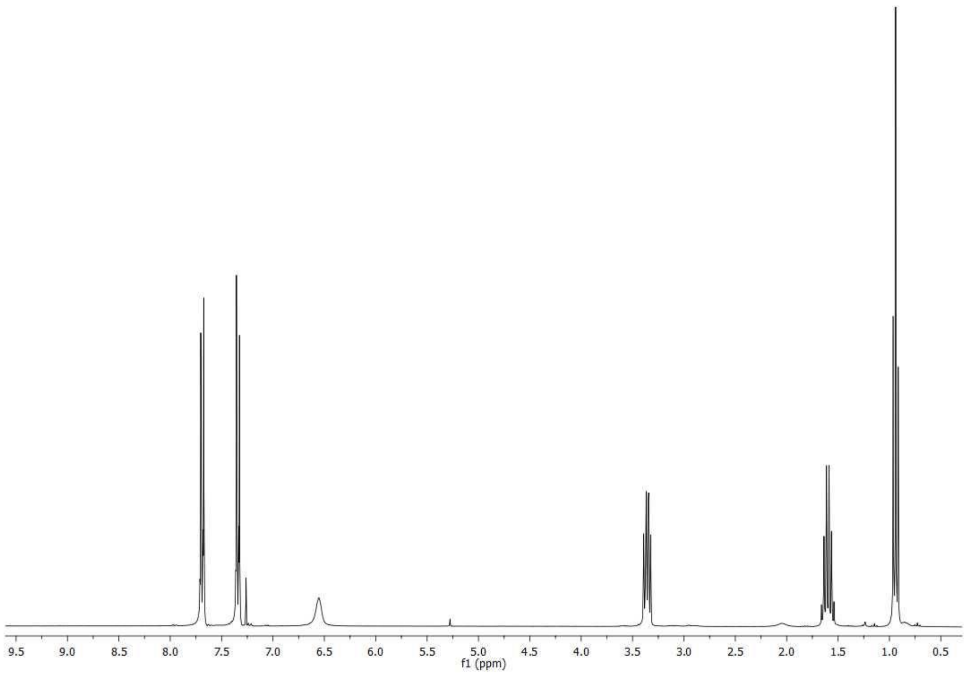

^{13}C NMR

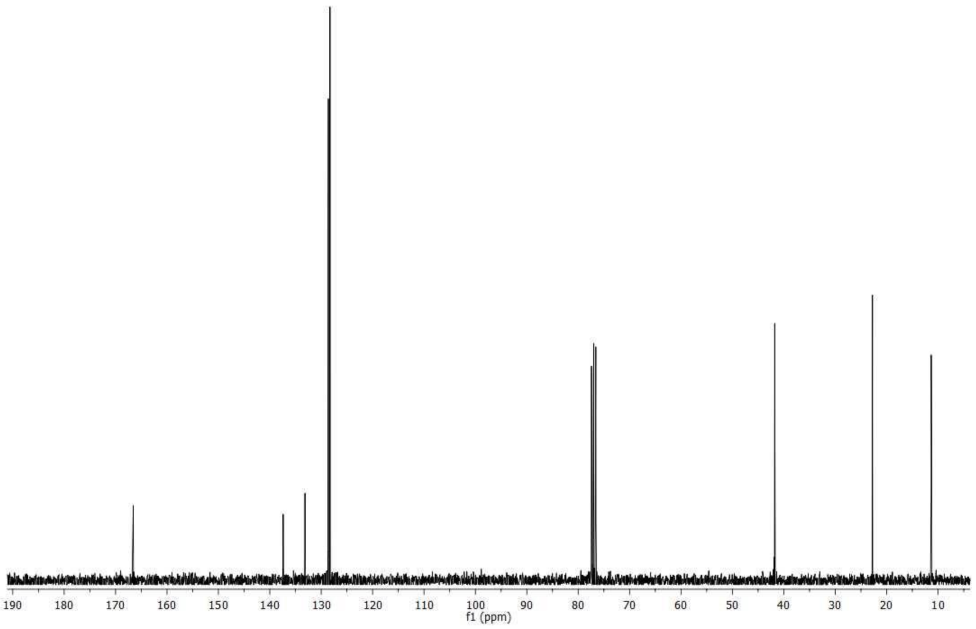

2-phenyl-*N*-propylacetamide (14)

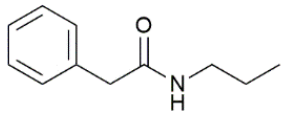

^{1}H NMR

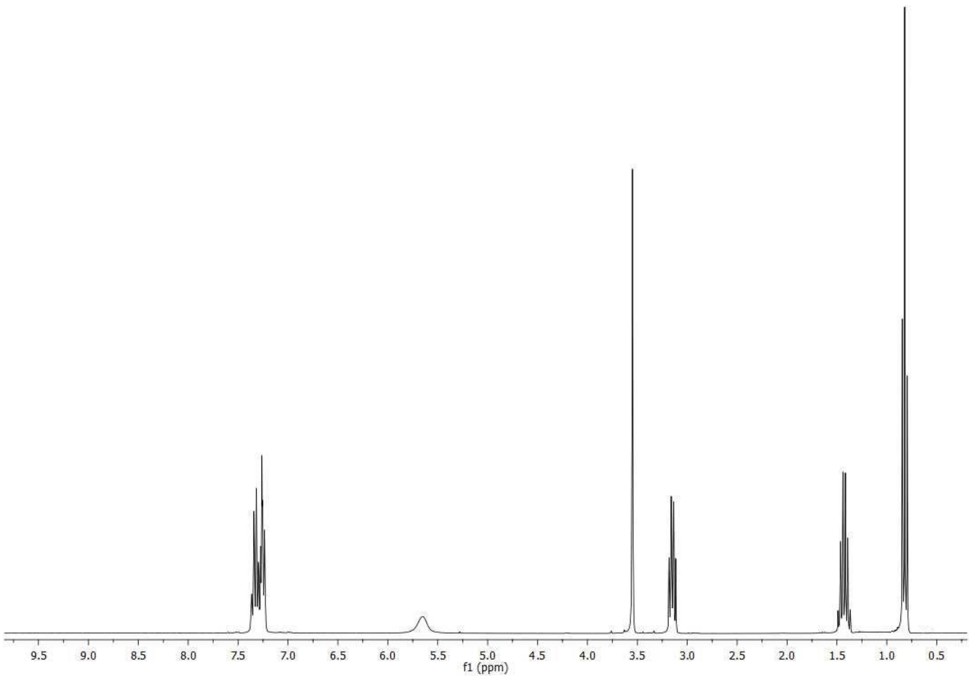

^{13}C NMR

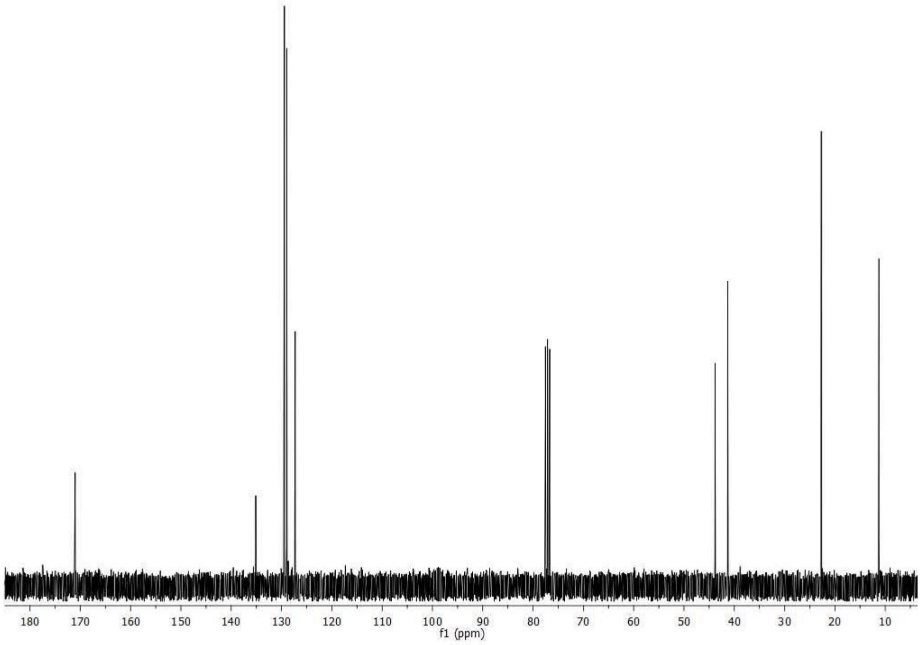

***N*-propylcinnamamide (15)**

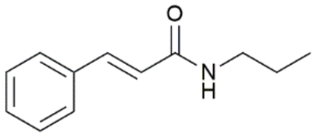

^{1}H NMR

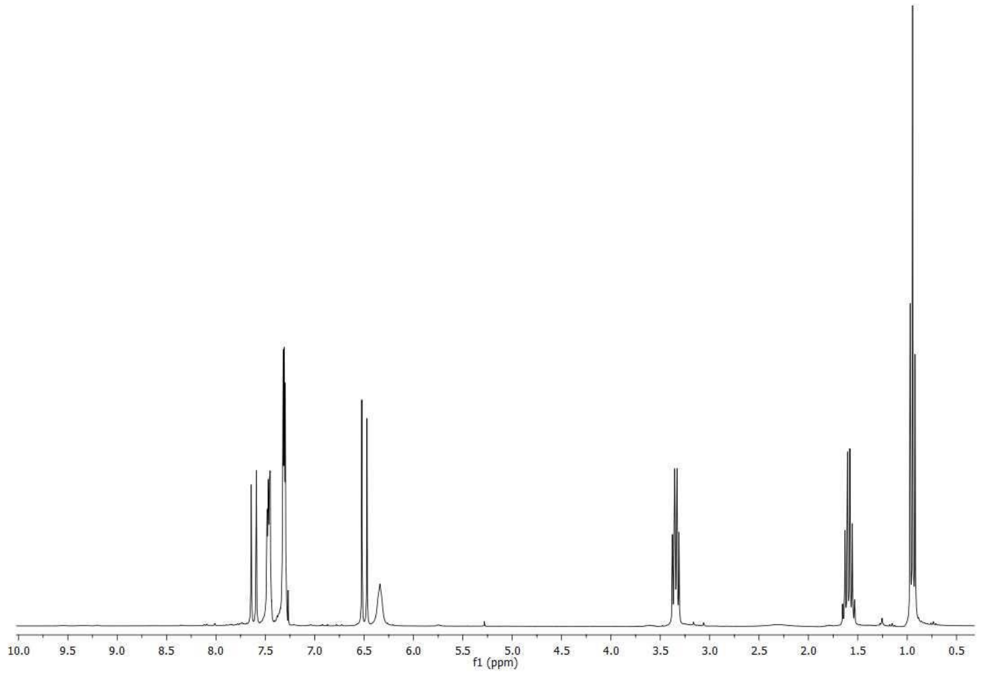

^{13}C NMR

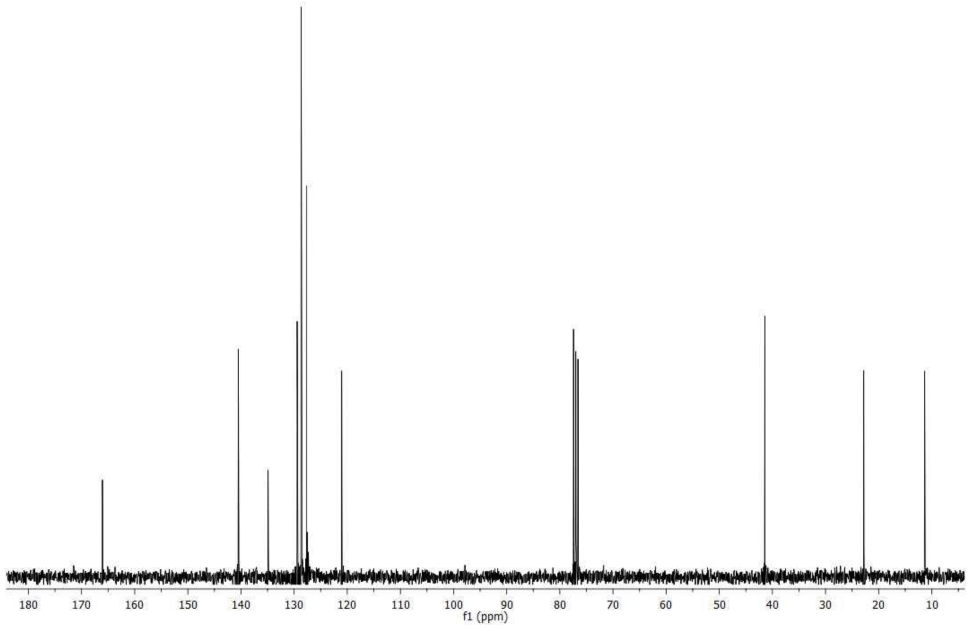

***N*-propylpalmitamide (16)**

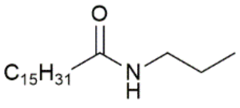

^{1}H NMR

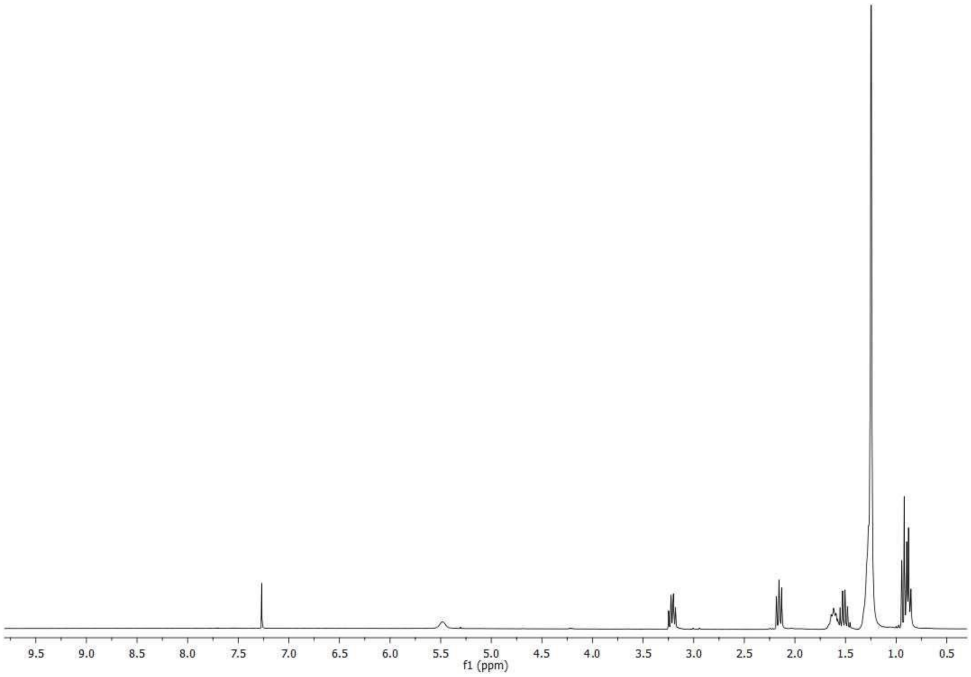

^{13}C NMR

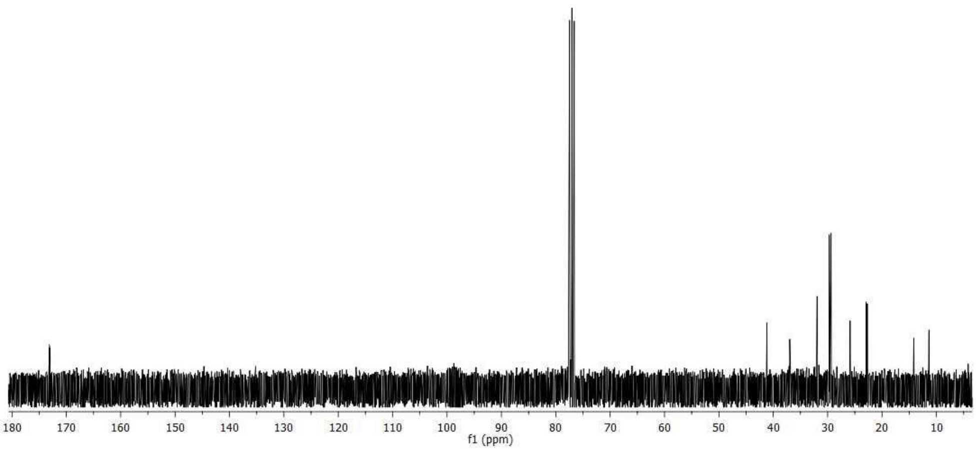

***N*,*N*-diethylbenzamide (17)**

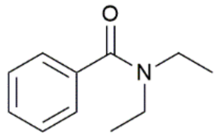

^{1}H NMR

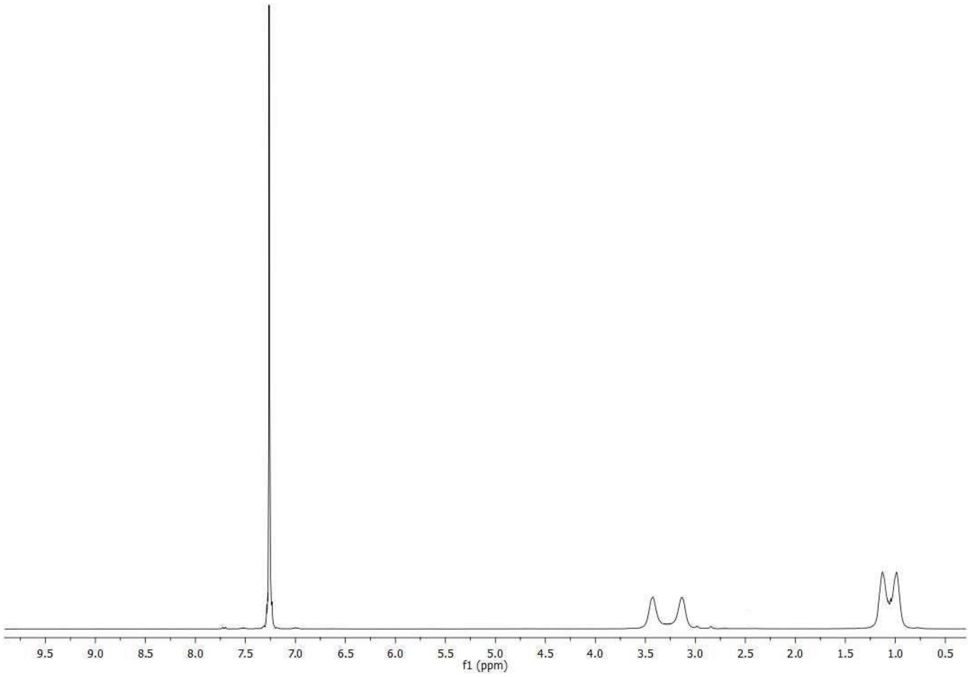

^{13}C NMR

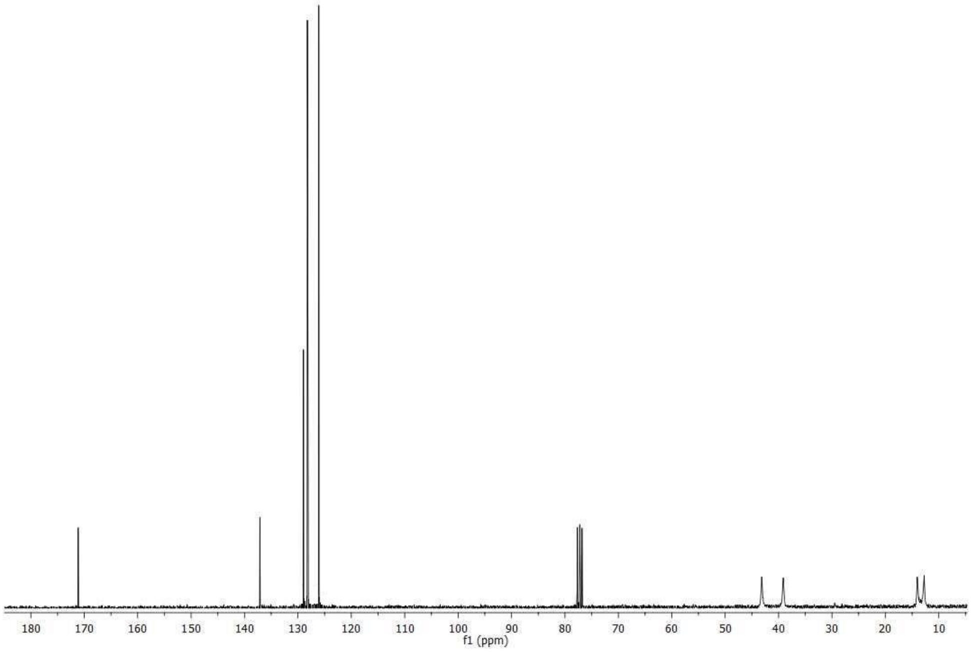

***N*,*N*-diethyl-4-nitrobenzamide (18)**

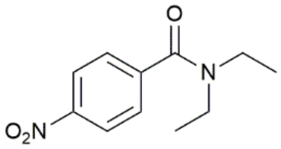

^{1}H NMR

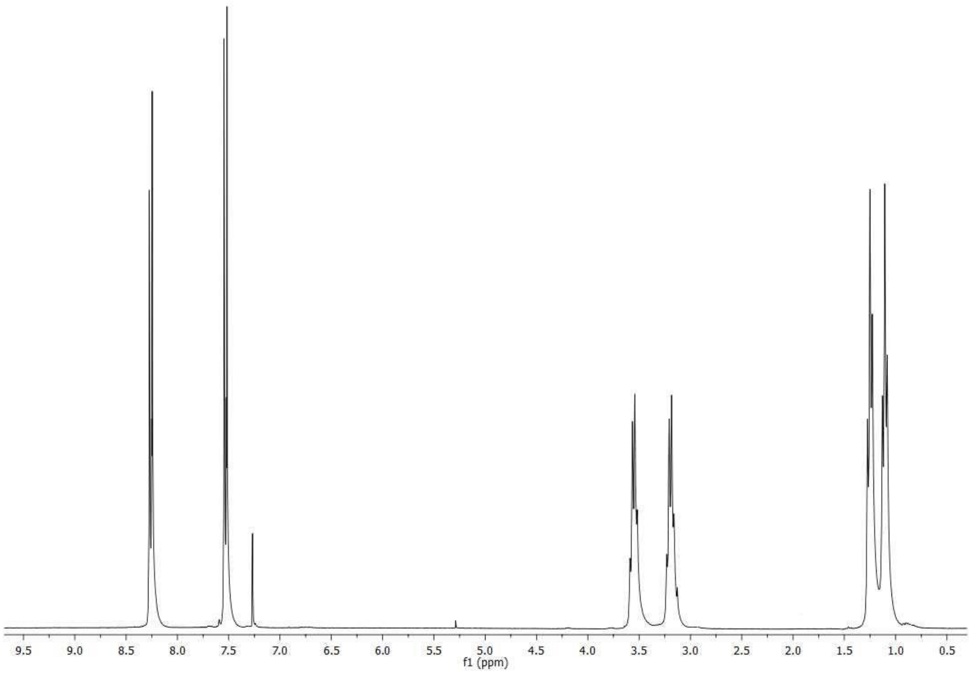

^{13}C NMR

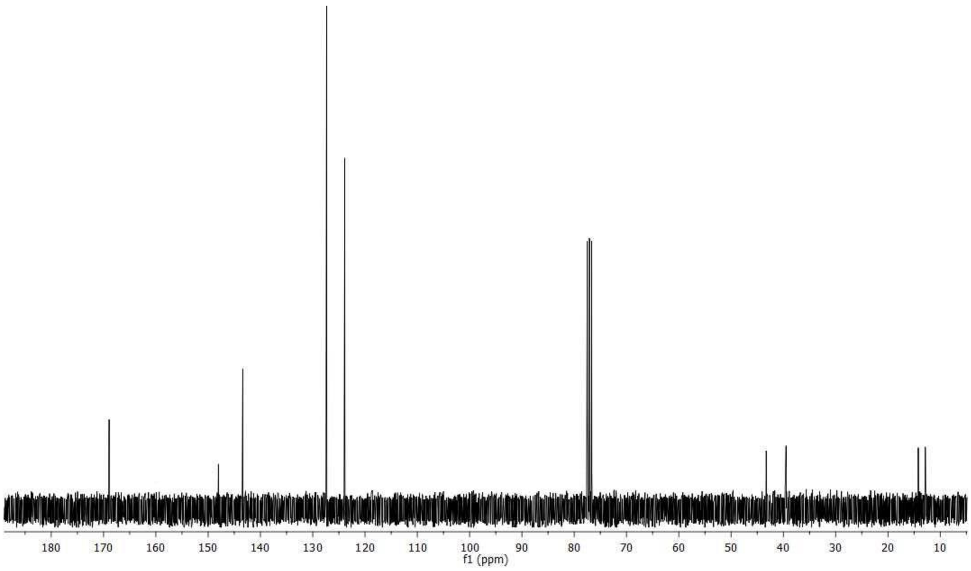

***N*,*N*-diethyl-4-methoxybenzamide (19)**

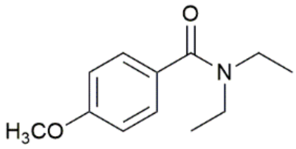

^{1}H NMR

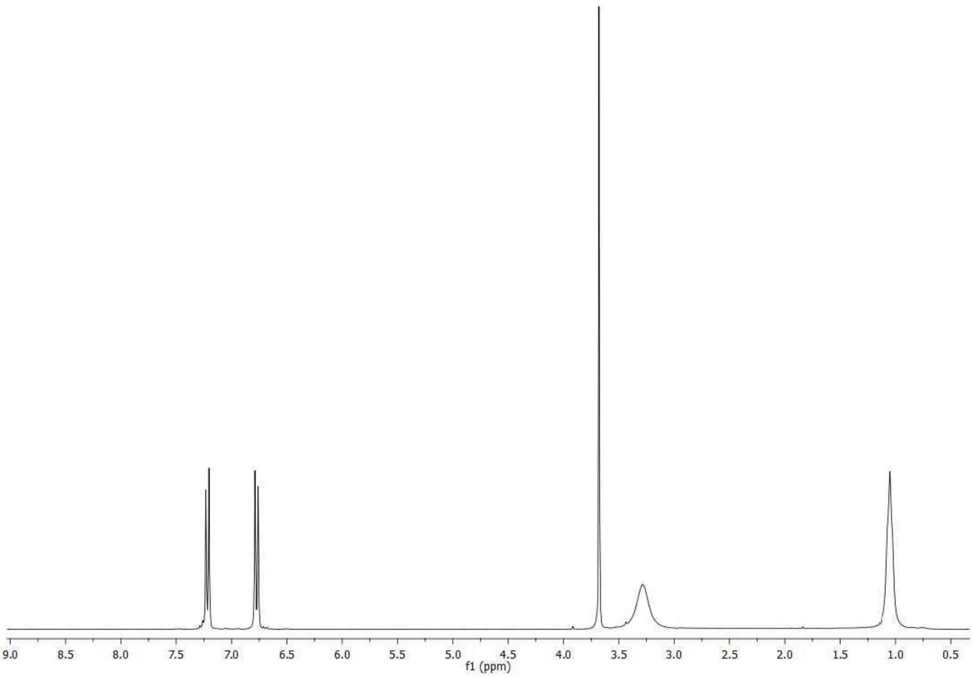

^{13}C NMR

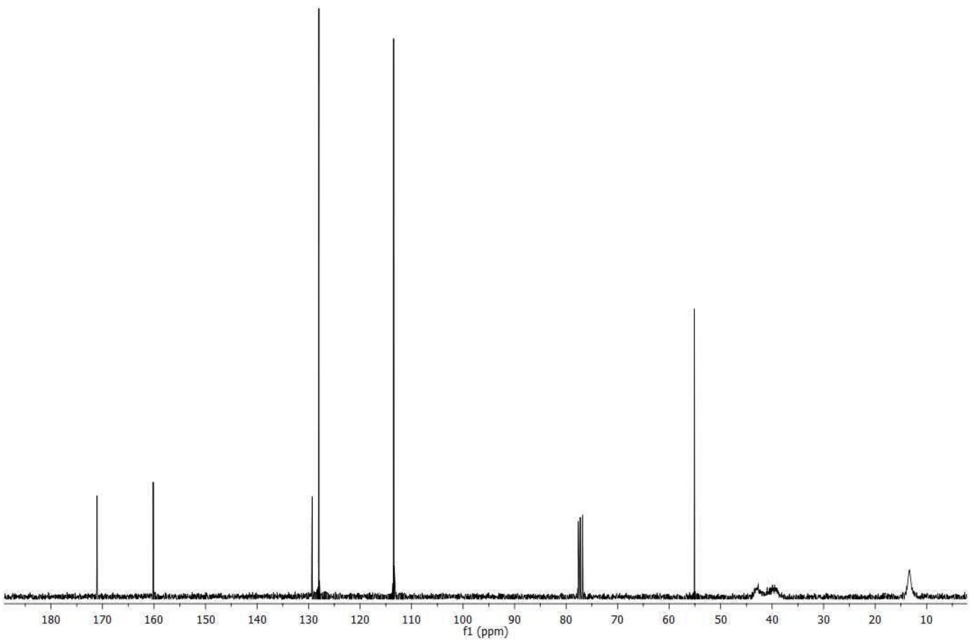

4-chloro-*N*,*N*-diethylbenzamide (20)

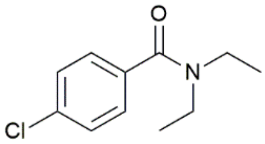

^{1}H NMR

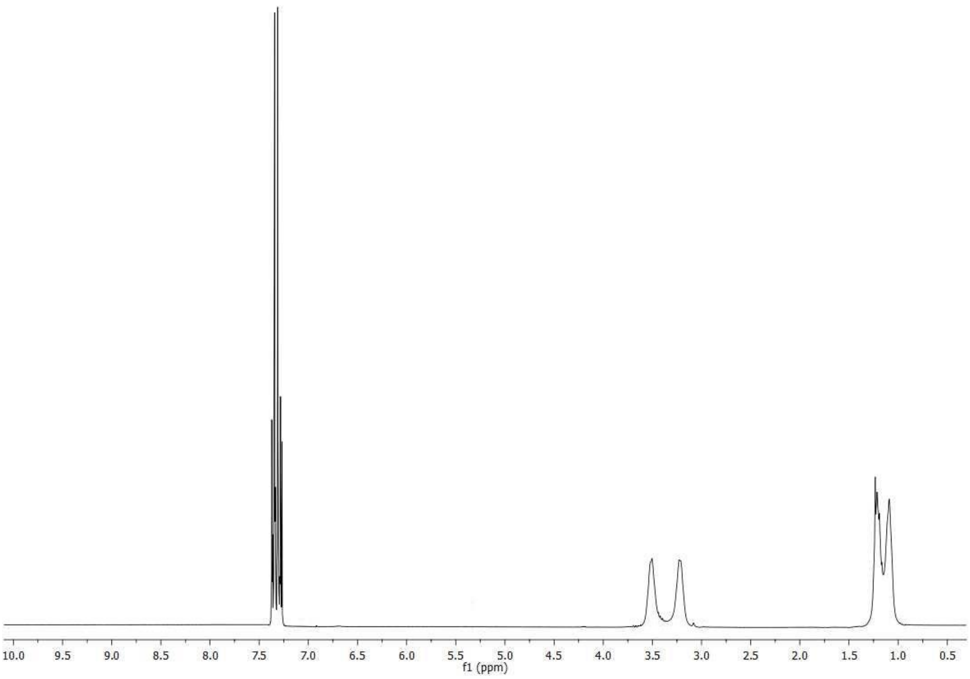

^{13}C NMR

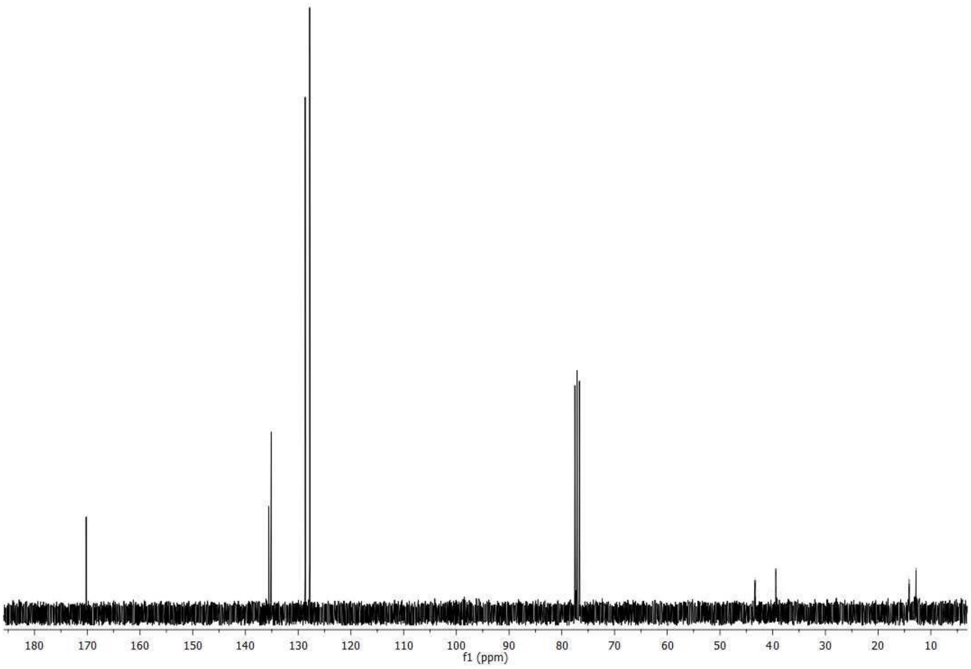

***N*,*N*-diethyl-2-phenylacetamide (21)**

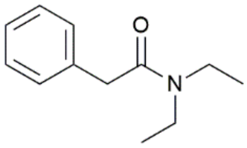

^{1}H NMR

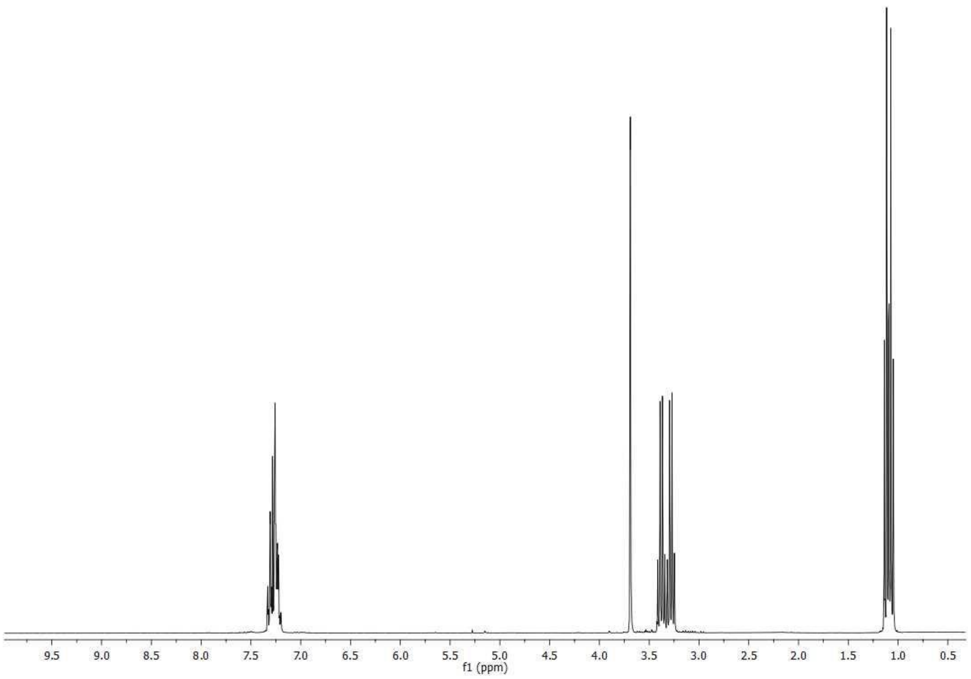

^{13}C NMR

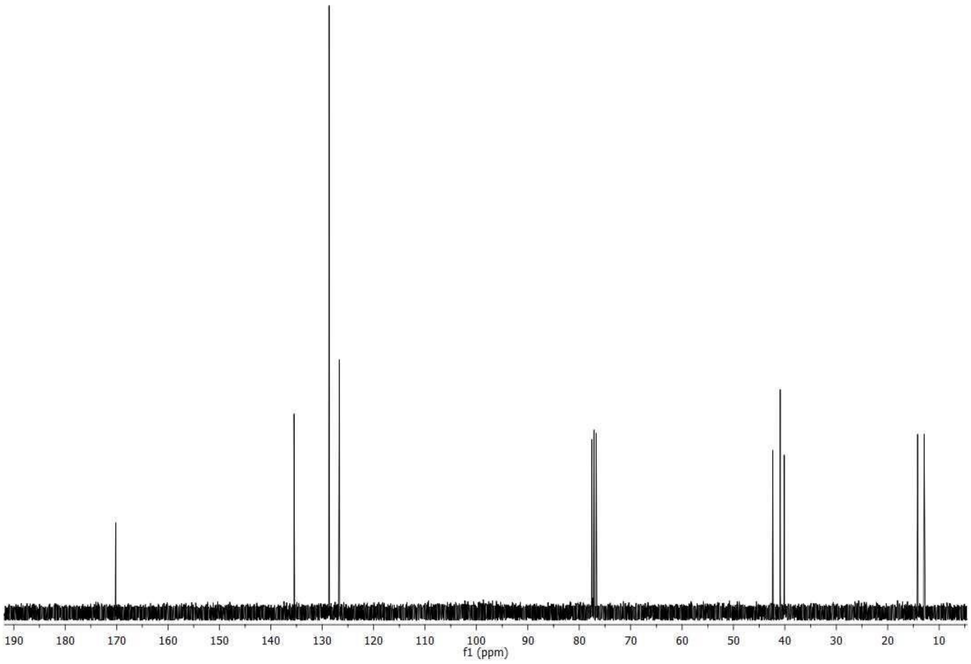

***N*,*N*-diethylcinnamamide (22)**

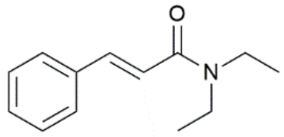

^{1}H NMR

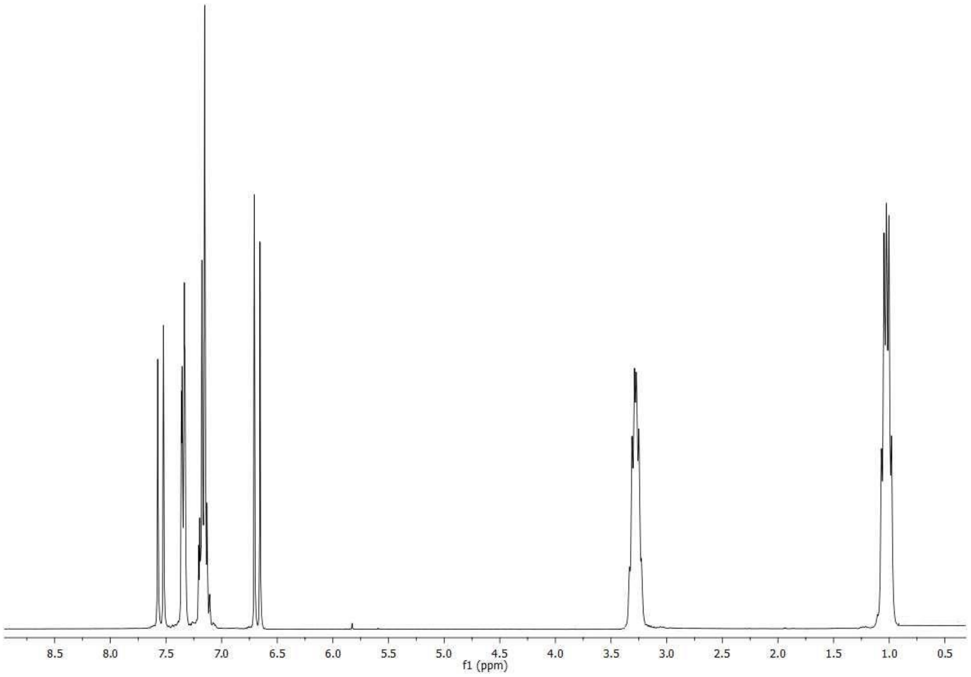

^{13}C NMR

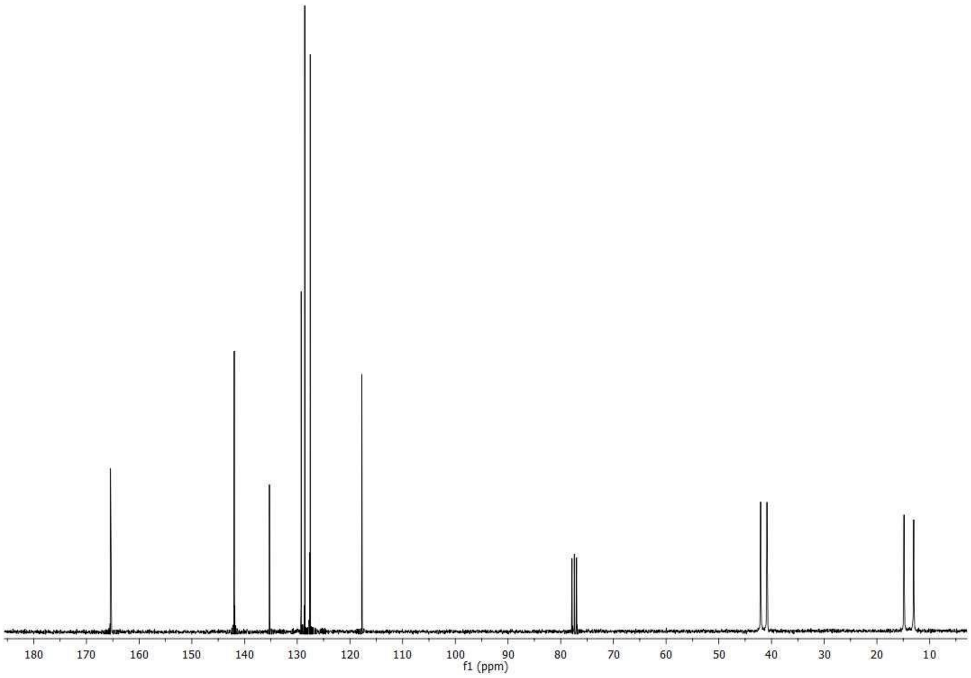

***N*,*N*-diethylpalmitamide (23)**

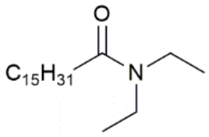

^{1}H NMR

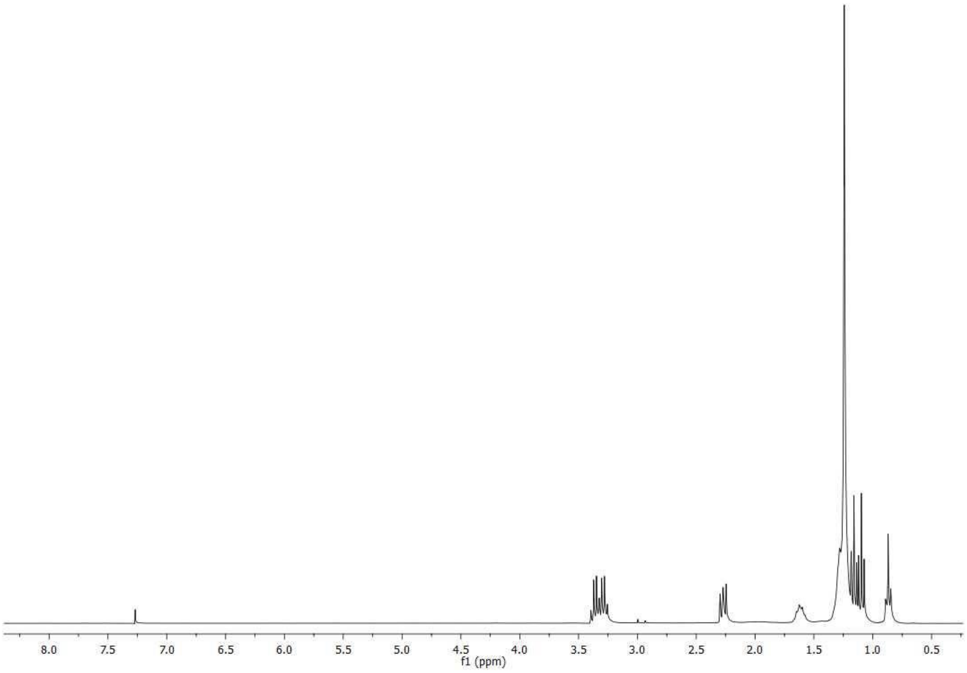

^{13}C NMR

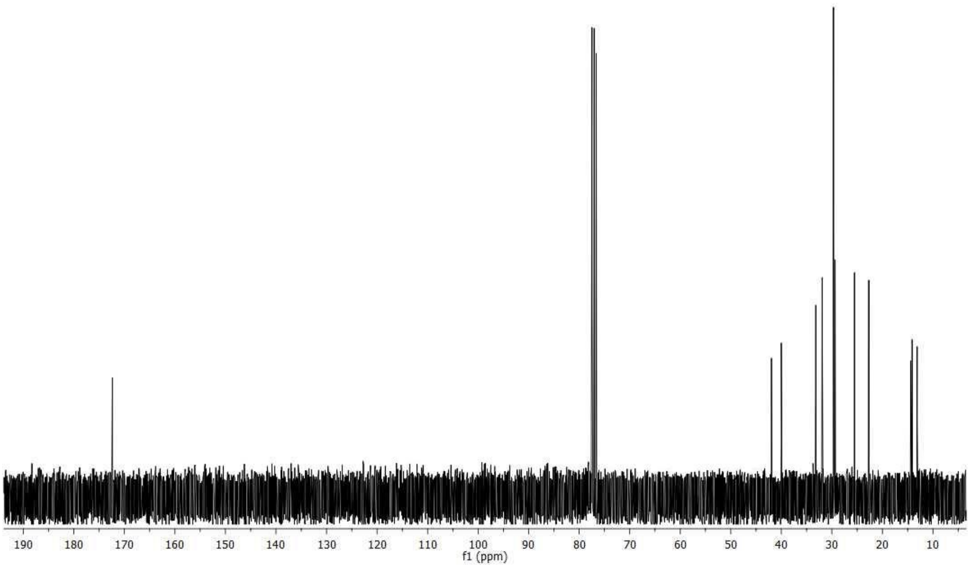

***N*-phenylpivalamide (24)**

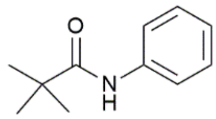

^{1}H NMR

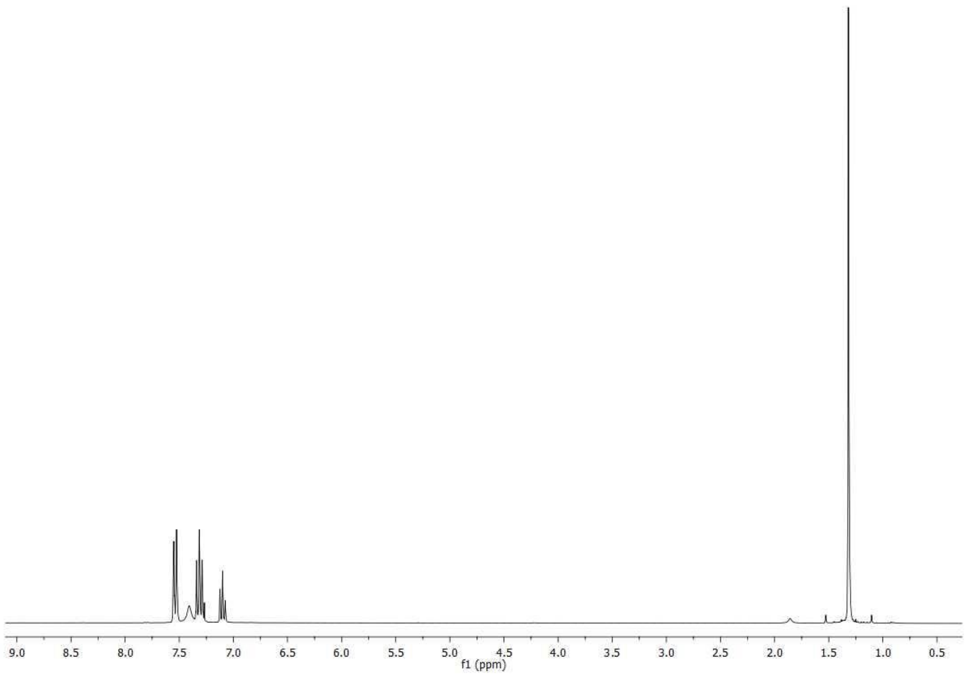

^{13}C NMR

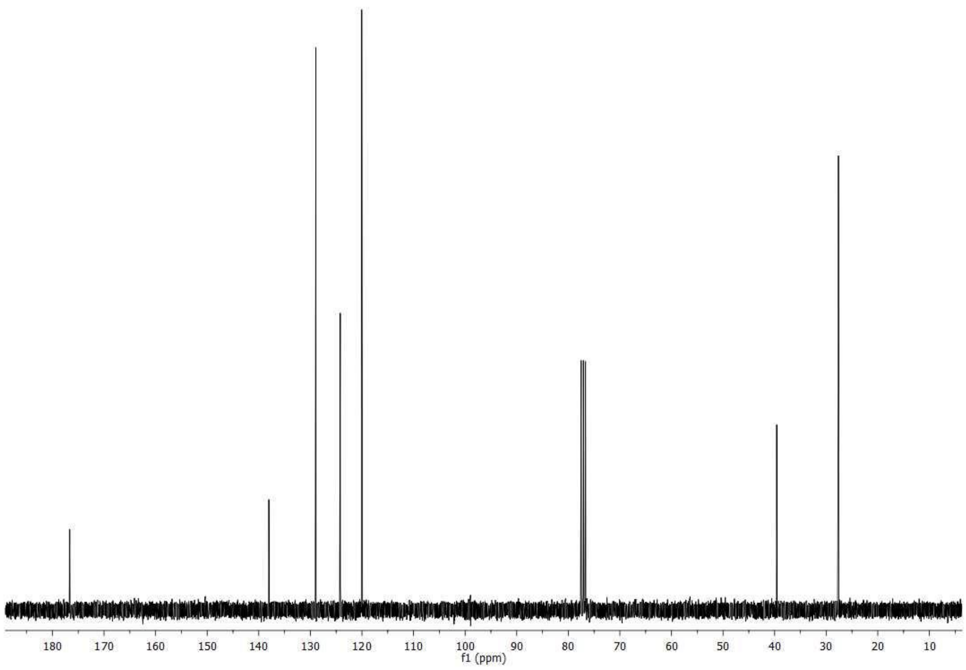

***N*-propylpivalamide (25)**

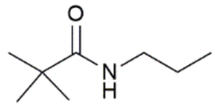

^{1}H NMR

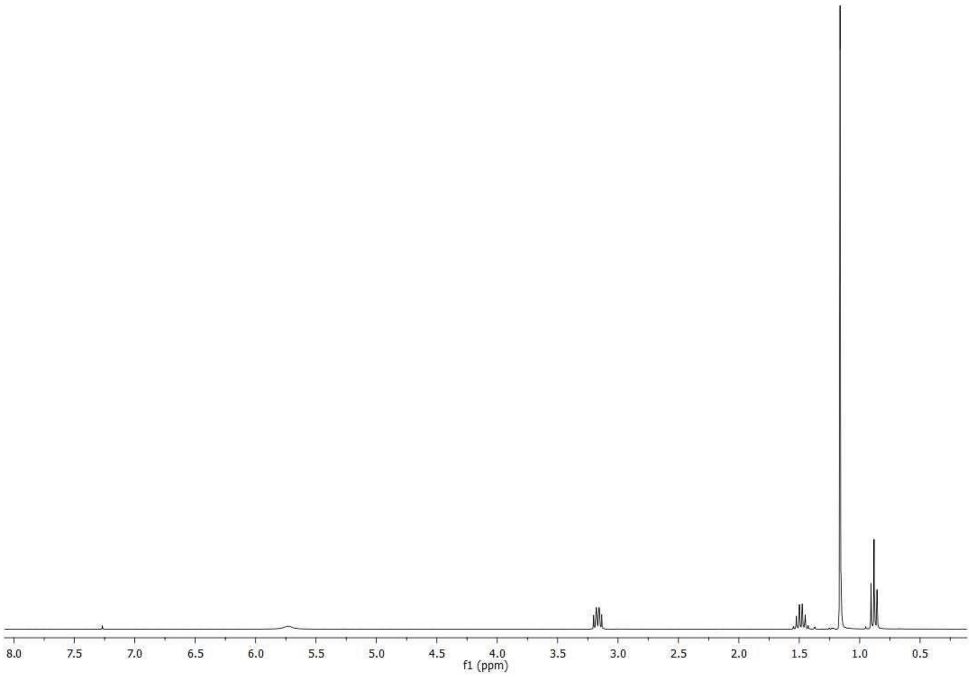

^{13}C NMR

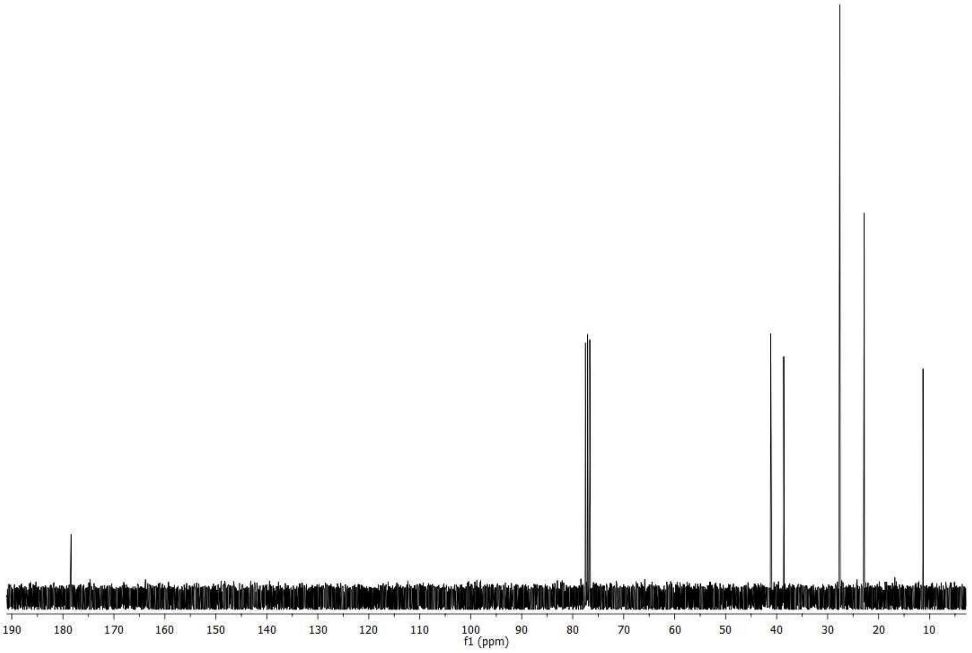

***N*,*N*-diethylpivalamide (26)**

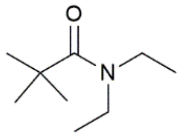

^{1}H NMR

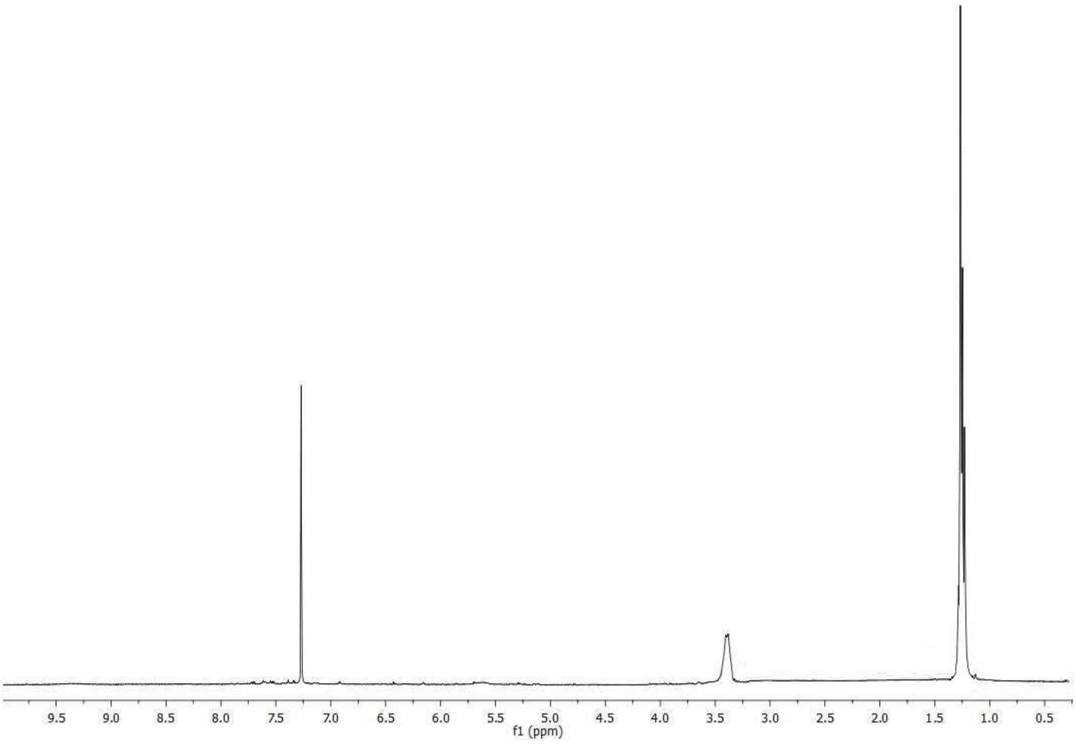

(*S*)-2-(*N*-*tert*-Butoxycarbonylamino)-*N*-phenylpropanamide (27)

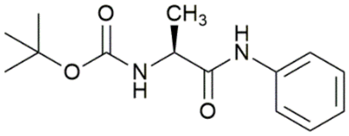

^{1}H NMR

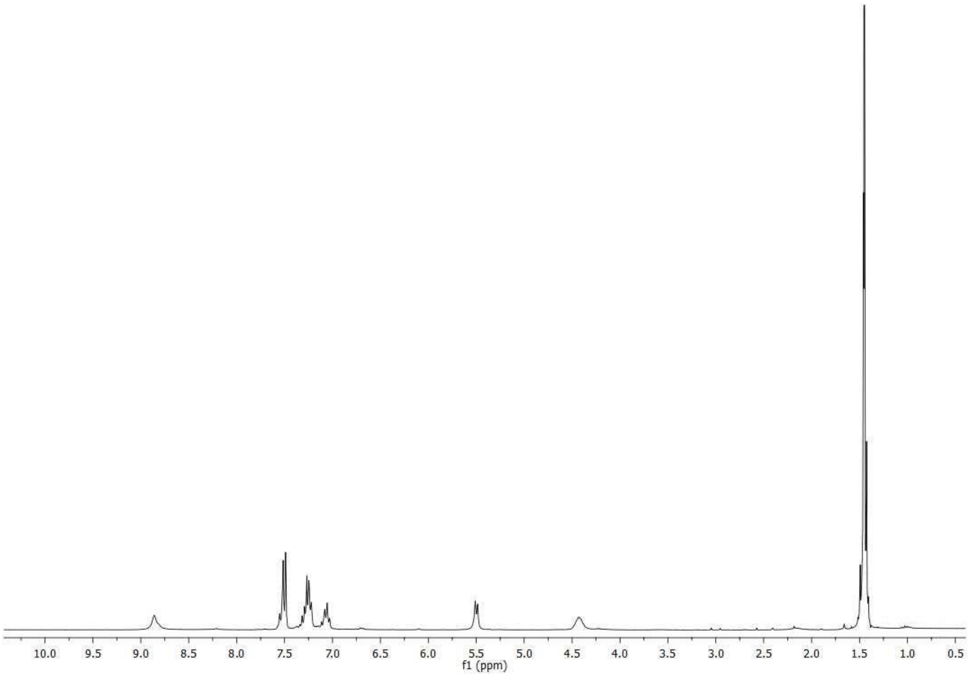

^{13}C NMR

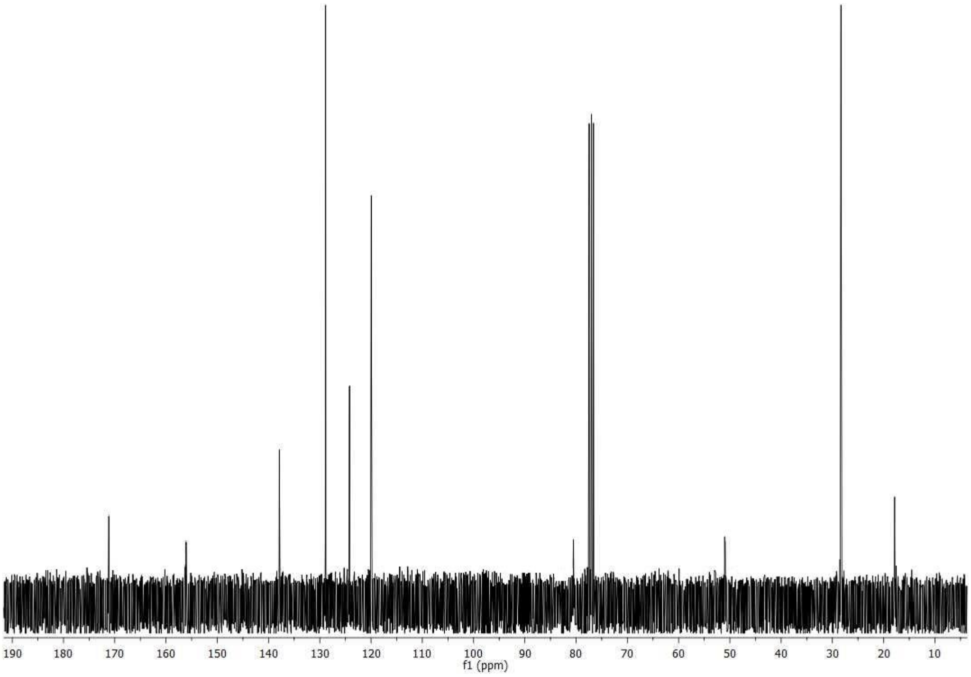

(*R*)-2-(*N*-*tert*-Butoxycarbonylamino)-*N*-phenylpropanamide (28)

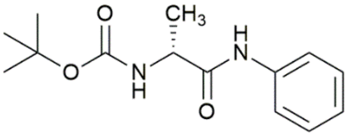

^{1}H NMR

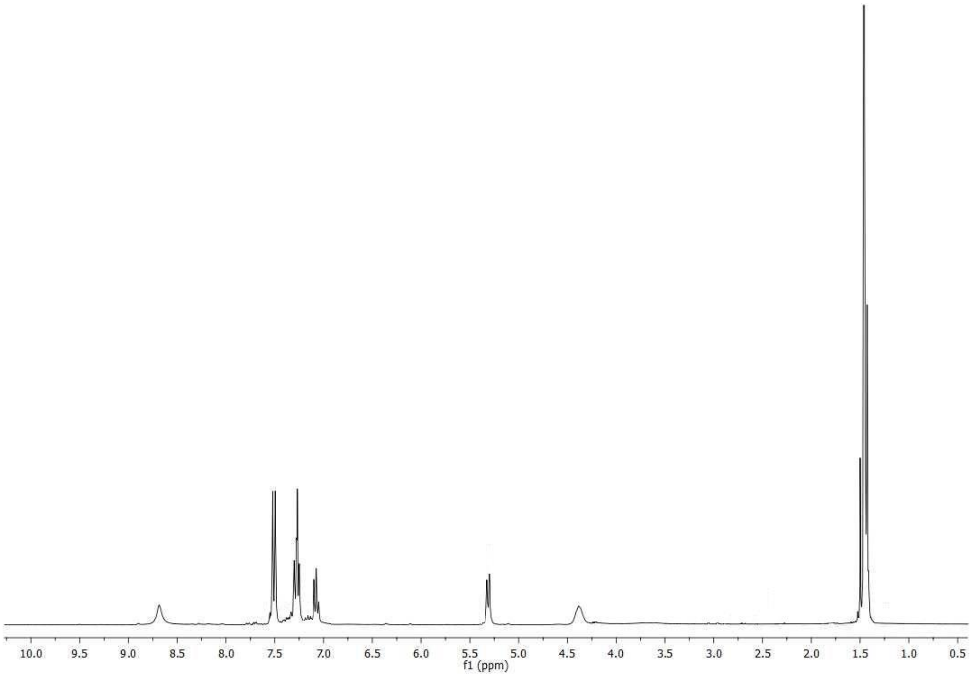

^{13}C NMR

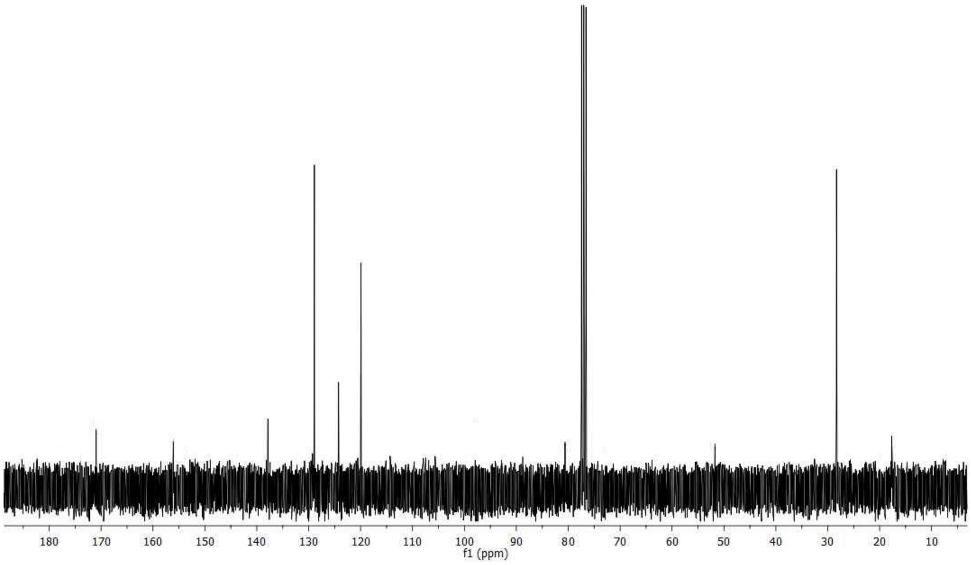

Supplement: Supplementary file 1 — Additional file 1. Supporting informations. [file 13065_2017_318_MOESM1_ESM.pdf]
